# Merkel cell polyomavirus recruits MYCL to the EP400 complex to promote oncogenesis

**DOI:** 10.1371/journal.ppat.1006668

**Published:** 2017-10-13

**Authors:** Jingwei Cheng, Donglim Esther Park, Christian Berrios, Elizabeth A. White, Reety Arora, Rosa Yoon, Timothy Branigan, Tengfei Xiao, Thomas Westerling, Alexander Federation, Rhamy Zeid, Benjamin Strober, Selene K. Swanson, Laurence Florens, James E. Bradner, Myles Brown, Peter M. Howley, Megha Padi, Michael P. Washburn, James A. DeCaprio

**Affiliations:** 1 Department of Medical Oncology, Dana-Farber Cancer Institute, Boston, Massachusetts, United States of America; 2 Department of Medicine, Brigham and Women’s Hospital, Boston, Massachusetts, United States of America; 3 Department of Medicine, Harvard Medical School, Boston, Massachusetts, United States of America; 4 Graduate School of Arts and Sciences, Harvard University, Boston, Massachusetts, United States of America; 5 Department of Microbiology and Immunobiology, Harvard Medical School; Boston, Massachusetts, United States of America; 6 Center for Functional Cancer Epigenetics, Dana-Farber Cancer Institute, Boston, Massachusetts, United States of America; 7 Stowers Institute for Medical Research, Kansas City, Missouri, United States of America; 8 Department of Biostatistics and Computational Biology, Dana-Farber Cancer Institute, Boston, Massachusetts, United States of America; 9 Department of Pathology and Laboratory Medicine, University of Kansas Medical Center, Kansas City, Kansas, United States of America; Fred Hutchinson Cancer Research Center, UNITED STATES

## Abstract

Merkel cell carcinoma (MCC) frequently contains integrated copies of Merkel cell polyomavirus DNA that express a truncated form of Large T antigen (LT) and an intact Small T antigen (ST). While LT binds RB and inactivates its tumor suppressor function, it is less clear how ST contributes to MCC tumorigenesis. Here we show that ST binds specifically to the MYC homolog MYCL (L-MYC) and recruits it to the 15-component EP400 histone acetyltransferase and chromatin remodeling complex. We performed a large-scale immunoprecipitation for ST and identified co-precipitating proteins by mass spectrometry. In addition to protein phosphatase 2A (PP2A) subunits, we identified MYCL and its heterodimeric partner MAX plus the EP400 complex. Immunoprecipitation for MAX and EP400 complex components confirmed their association with ST. We determined that the ST-MYCL-EP400 complex binds together to specific gene promoters and activates their expression by integrating chromatin immunoprecipitation with sequencing (ChIP-seq) and RNA-seq. MYCL and EP400 were required for maintenance of cell viability and cooperated with ST to promote gene expression in MCC cell lines. A genome-wide CRISPR-Cas9 screen confirmed the requirement for MYCL and EP400 in MCPyV-positive MCC cell lines. We demonstrate that ST can activate gene expression in a EP400 and MYCL dependent manner and this activity contributes to cellular transformation and generation of induced pluripotent stem cells.

## Author summary

Merkel cell carcinoma (MCC) is a highly aggressive, neuroendocrine cancer of the skin. MCC frequently contains integrated copies of Merkel cell polyomavirus DNA and expresses two viral transcripts including a truncated form of Large T antigen (LT) and an intact Small T antigen (ST). While LT binds the Retinoblastoma protein and inactivates its tumor suppressor function, it is less clear how ST contributes to MCC tumorigenesis. Here we show that ST specifically recruits the MYC homolog MYCL (L-MYC) to the 15-component EP400 histone acetyltransferase and chromatin remodeling complex. The ST-MYCL-EP400 complex binds to specific gene promoters to activate their expression. Both MYCL and EP400 are required for maintenance of MCC cell line viability and can cooperate with ST to promote gene expression. We demonstrate that ST enhances the interaction between MYCL and the EP400 complex interaction and this activity contributes to transcriptional activation, oncogenesis and reprogramming of MCC.

## Introduction

Merkel cell carcinoma (MCC) is an aggressive skin cancer with a high rate of mortality. Risk factors for developing MCC include immunosuppression and UV-induced DNA damage from excessive exposure to sunlight [1]. Recognition of the immunosuppressive risk for MCC prompted a search to identify pathogens and led to the discovery of Merkel cell polyomavirus (MCPyV) [2]. MCPyV-positive MCC tumors contain clonally integrated copies of viral DNA and express small T antigen (ST) and a truncated form of large T antigen (LT). Genome sequencing of virus-negative MCC revealed an extremely high number of single nucleotide polymorphisms containing the C>T transition consistent with UV damage [3, 4]. In contrast, MCPyV positive tumors contain very few somatic mutations suggesting that MCPyV ST and LT contribute the major oncogenic activity to MCC development. In all virus-positive MCC cases reported to date, LT has undergone truncations that disrupt viral replication activities but leave the LXCXE, RB-binding, motif intact [5]. While LT can bind and inactivate RB, it is less clear how ST contributes to MCC tumorigenesis [6, 7].

MCPyV ST has oncogenic activity and can contribute to tumor development in mouse models [8, 9]. Similar to ST from other polyomaviruses, MCPyV ST can bind to the A and C subunits of protein phosphatase 2A (PP2A) [10]. However, ST binding to PP2A may not be necessary for its oncogenic activity. A unique domain of ST known as the LT stabilizing domain (LSD) has been reported to bind FBXW7 and CDC20, functions to increase levels of LT, and contributes to its transforming function [11, 12]. ST can activate MTOR signaling and promote the phosphorylation of EIF4EBP1 (4EBP1) [13]. ST can also perturb NFκB and inflammatory signaling [14, 15]. When expressed in fibroblasts, ST led to increased levels of glycolytic genes including the lactate transporter SLC16A1 (MCT1) and induction of aerobic glycolysis also known as the Warburg effect [16]. However, it is not clear how ST contributes to so many distinct activities.

The EP400 histone acetyltransferase complex is involved in multiple biological events including transcription, stem cell maintenance and DNA damage response. The mammalian EP400 complex contains at least 15 distinct components including the large subunits EP400 (also known as p400) and TRRAP plus ACTL6A, BRD8, DMAP1, EPC1 (and its homologue EPC2), ING3, KAT5 (also known as Tip60), MBTD1, MEAF6, MORF4L1 (and MORFL2), MRGBP, RUVBL1 (and RUVBL2), VPS72 and YEATS4 [17–20]. The EP400 complex contains several intrinsic enzymatic activities including EP400 chaperone activity for histone variants H3.3 and H2AZ, KAT5 mediated acetylation of histones H2A and H4, and the DNA helicase activity of RUVBL1 and RUVBL2. TRRAP can bind directly to MYC and has been reported to bind equally well to the homologue MYCN and poorly to MYCL (L-MYC) [21, 22].

Here we demonstrate that MCPyV ST recruits MYCL to the EP400 complex to activate specific gene expression, promote cellular transformation and contribute to its oncogenic potential.

## Results

### MCPyV ST binds MYCL and the EP400 complex

**Fig 1:**
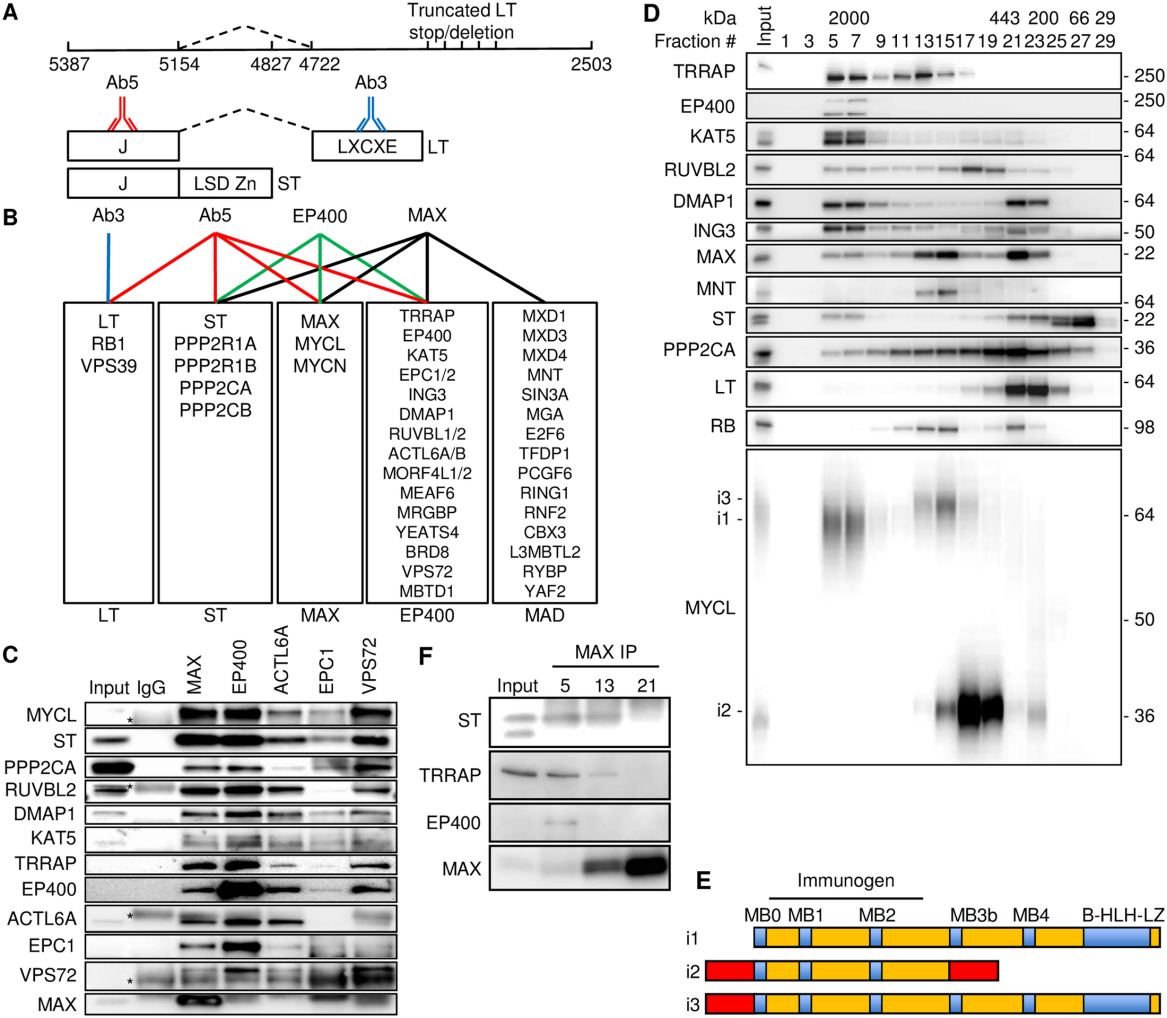
MCPyV ST binds MYCL and EP400 complex. (A) MCPyV early region showing nucleotide positions for LT start (5387), ST stop (4827), LT stop (2503), and LT splice donor (5154) and acceptor (4722) and approximate positions of mutations that result in truncated LT found in MCC. LT and ST share an N-terminal J domain. The ST unique domain contains the LSD and Zn fingers. LT splices from J domain to a second exon containing the LXCXE or RB1 binding motif. Antibody Ab3 binds LT only and Ab5 binds both LT and ST. (B) Identification of co-precipitating proteins by MudPIT with antibodies Ab3 (LT, blue), Ab5 (LT/ST, red), EP400 (green) and MAX (black). See S1 Table for details. (C) MKL-1 lysates were immunoprecipitated (IP) with indicated antibodies (top) followed by immunoblotting with indicated antibodies (left). Asterisks indicate non-specific bands in IgG control immunoprecipitation lane. (D) MKL-1 lysates (Input) were separated in a Superose 6 column and fractions (#) were blotted with antibodies indicated on left. Protein size markers in kDa indicated at top and right. (E) Three MYCL isoforms (i1, i2, i3) are indicated (see also S1 Fig). Immunogen of MYCL antibody contained MYCL-i1 residues 16–139. (F) Fractions #5, 13 and 21 from Fig 1D were immunoprecipitated with MAX antibody and blotted.

To understand how MCPyV T antigens contribute to MCC oncogenesis, we performed a large-scale immunoprecipitation with a monoclonal antibody (Ab5) specific for the shared N-terminal region of LT and ST to identify associated cellular proteins from lysates of virus-positive MCC cell lines MKL-1 and WaGa (Fig 1A) [23]. Identification of the immunoprecipitated proteins by multi-dimensional protein identification technology (MudPIT) revealed MCPyV LT and ST (Fig 1B and S1 Table) [24]. RB1 and VPS39 were detected as expected given their previously reported association with LT [5, 25]. Both homologues of the PP2A scaffold (PPP2R1A, PPP2R1B) and catalytic (PPP2CA, PPP2CB) subunits were also detected, likely due to association with ST [10, 14]. Unexpectedly, Ab5 also co-precipitated MYCL and MAX as well as all known subunits of the EP400 complex listed above including ACTL6B, a homologue of ACTL6A, and the recently reported MBTD1 [20]. In contrast, MudPIT using Ab3, specific for LT only, identified LT, RB1 and VPS39 and none of the components of the EP400 complex.

To validate the interactions with endogenous proteins, MKL-1 cell lysates were immunoprecipitated with antibodies to MAX, EP400, ACTL6A, EPC1 and VPS72. Each of these antibodies co-precipitated ST, PPP2CA and MYCL as well as several components of the EP400 complex (Fig 1C). MudPIT with antibodies for EP400 identified all 15 subunits including homologs of the EP400 complex plus MYCL, MAX, ST and PP2A (Fig 1B and S1 Table). MudPIT with antibodies for MAX enriched for MYCL, ST, PP2A, all components of the EP400 complex plus several MAD and MAD-associated proteins [26, 27]. MudPIT with an IgG control antibody detected small amounts of RUVBL1, RUVBL2, MEAF6 and ACTL6B but none of the other EP400 complex components. Therefore, antibodies for MAX, EP400 and MCPyV ST each specifically co-precipitated MYCL, the EP400 complex, ST and PP2A (Fig 1B and S1A Fig).

To determine if ST could form a single complex with MYCL and the EP400 complex, we performed gel filtration of MKL-1 cell lysates [28]. Fractions #5–7 contained protein complexes of ≥ 2 MDa with ST, MYCL, MAX, several EP400 complex components and EP400 itself (Fig 1D). EP400 was only detected in the large complex fraction while other subunits of the complex including TRRAP, KAT5, RUVBL2, DMAP1 and ING3 were present in the large complex and in fractions with smaller sized complexes. Of note, MYCL1 isoform 1 (i1) was present in the ST-containing fractions #5–7 whereas the larger MYCL i3 was detected in intermediate sized fractions and the shortest form (i2) in smaller size fractions (Fig 1D and 1E, S1B and S1C Fig). An immunoprecipitation for MAX with lysates from fraction #5 co-precipitated EP400, TRRAP and ST (Fig 1F). In contrast, MAX co-precipitated TRRAP and ST but not EP400 from fraction #13 and neither TRRAP or EP400 from fraction #21. This indicates that a specific fraction of MAX binds to EP400, a key component of the ST-MYCL complex [29].

### MCPyV ST binds specifically to the EP400 complex

**Fig 2:**
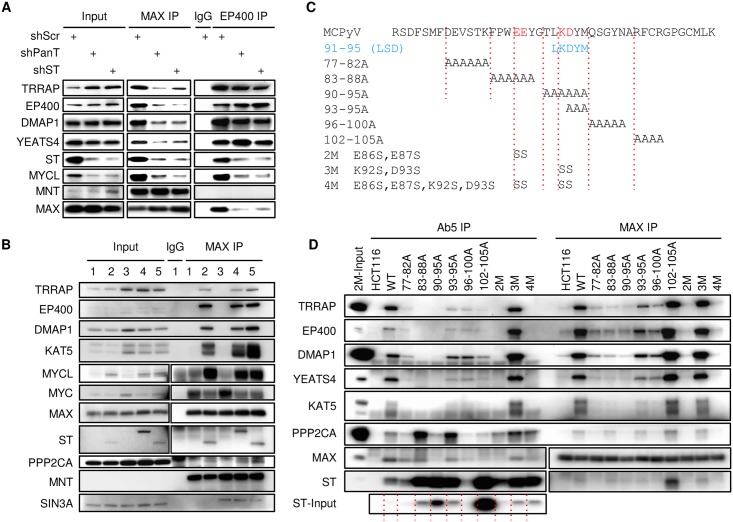
MCPyV ST binds specifically to EP400 complex. **(A)** MKL-1 cells transduced with lentiviral scramble shRNA (shScr) or shRNA specific for LT and ST (shPanT) or ST only (shST) for 1 day followed by selection in puromycin (1 μg/ml) for additional 3 days were lysed (Input) and immunoprecipitated for MAX, EP400 or non-specific IgG and blotted. (B) Lysates from HCT116 (lanes 1 and 2) or UISO (lanes 3, 4 and 5) cells stably expressing MCPyV ST (lanes 2 and 5) or a C-terminal epitope tagged ST (lane 4) were immunoblotted (Input) or immunoprecipitated with antibodies to MAX or non-specific IgG. See also S2A Fig. (C) MCPyV ST residues 70–112 is shown with corresponding substitution mutations. See also S2C Fig. Residues in the LT stabilization domain (LSD) are indicated [10, 11]. (D) HCT116 cells stably expressing wild type (WT) MCPyV ST or indicated mutant constructs. Lysates were immunoprecipitated with Ab5 (ST) or MAX antibodies and blotted. Red dashed lines are shown to indicate 11 lanes in ST-Input lanes. Identical panel is also shown in S2D Fig for input.

To determine the contribution of MCPyV ST binding to MYCL and the EP400 complex in MCC, we transduced MKL-1 cells with lentiviral shRNAs targeting both LT and ST (shPanT) or ST only (shST) [13, 30]. Expression of either shRNA but not scrambled shRNA (shScr) led to reduced levels of ST and MYCL (Fig 2A, S2A Fig). Reduced levels of ST led to decreased ability of MAX to co-precipitate EP400, TRRAP, DMAP1 and YEATS4 and reduced the ability of EP400 to co-precipitate MYCL and MAX. Of note, when ST levels were reduced, EP400 retained the ability to bind to other components of the EP400 complex including TRRAP, DMAP1 and YEATS4.

To determine if MCPyV ST could increase the ability of MAX to bind to the EP400 complex, we introduced ST or C-terminal HA-tagged ST into HCT116 cells and a virus-negative MCC cell line UISO. Immunoprecipitation for MAX from parental HCT116 and UISO cell lysates readily co-precipitated MYC but not any EP400 complex components (Fig 2B). However, in the presence of MCPyV ST, MAX efficiently co-precipitated TRRAP, EP400, DMAP1 and KAT5. Of note, MYCL levels increased in HCT116 and UISO cells when ST was expressed (Fig 2B, Input). Stable expression of ST in primary human foreskin fibroblasts (HFF) also increased levels of MYCL (S2B Fig).

To determine if ST interaction with the EP400 complex was specific, we generated a series of ST mutants and stably expressed them in HCT116 cells. We focused the mutagenesis on a region of MCPyV ST within the unique domain that is not well conserved with ST from other human polyomaviruses and includes the LSD motif (residues 91–95) (Fig 2C, S2C Fig). Immunoprecipitation for ST containing alanine substitutions of residues 83 to 88 (83-88A) showed decreased binding to EP400 complex components while retaining strong binding to PP2A (Fig 2D, S2D Fig). Within this region, substitution of E86 and E87 with serine (E86S, E87S referred to as 2M) led to reduced EP400 complex binding yet retained some PP2A binding. In contrast, alanine substitution of residues 83 to 95 (83-88A, 90-95A, 93-95A) or 102 to 105 (102-105A) led to increased levels of ST relative to WT ST (Fig 2D, S2D Fig). Within this region, substitution of K92 and D93 with serine (K92S, D93S, referred to as 3M) led to increased levels of MCPyV ST (Fig 2D, S2D Fig). Combining the 2M and 3M mutants to create 4M (E86S, E87S, K92S, D93S) resulted in a ST construct that expressed at levels higher than WT ST and retained PP2A binding, but was unable to co-precipitate the EP400 complex. To test the ability of these ST constructs to promote MAX binding to the EP400 complex, we performed an IP with MAX antibodies. WT, 102-105A, and 3M ST led to increased ability of MAX to co-precipitate the EP400 complex and PP2A, while the 2M and 4M mutants were not co-precipitated by MAX and did not enable MAX to co-precipitate the EP400 complex or PP2A (Fig 2D).

### ST requires MYCL and MAX heterodimers to sustain MCC viability

**Fig 3:**
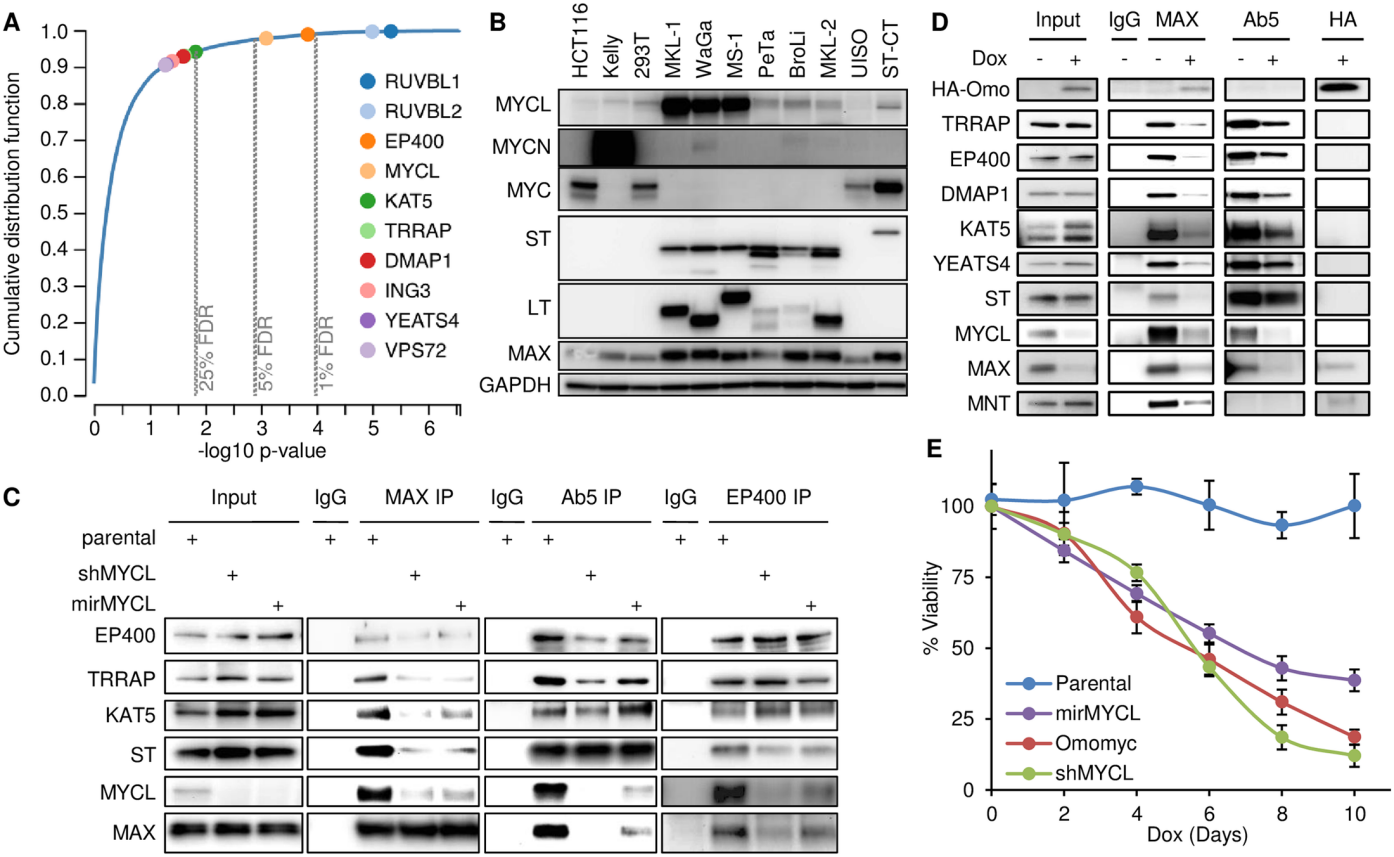
ST requires MYCL to sustain MCC viability. (A) CRISPR-Cas9 screen of MKL-1 cells was analyzed in the MAGeCK-VISPR pipeline. Cumulative distribution function of p-values plotted based on 18,493 human genes. EP400 complex components and MYCL were identified in CRISPR screen negative selection with p-values < 0.05 were indicated. See also S3A–S3C Fig and S2 Table. (B) Lysates from virus-positive MCC cell lines MKL-1, WaGa, MS-1, PeTa, BroLi and MKL-2, virus-negative MCC cell line UISO, and additional lines were immunoblotted. ST-CT are UISO cells stably expressing C-terminal epitope tagged ST. (C) Lysates from MKL-1 cell lines containing Dox-inducible shRNA (shMYCL) or miRNA (mirMYCL) specific for MYCL, prepared 2 days after addition of 0.3 μg/ml Dox (Input), were immunoprecipitated for MAX, Ab5, EP400 or non-specific IgG and blotted. (D) MKL-1 cells containing Dox-inducible HA tagged Omomyc before (-) or after (+) 5 days of Dox treatment. Dox (0.3 μg/ml) was added every two days. Lysates (Input) were immunoprecipitated with non-specific IgG, MAX, Ab5 and HA antibodies and blotted. (E) Viability of MKL-1 Dox-inducible cell lines described in C and D. 3,000 cells of each line were aliquoted in 96 well plate on day 0. Total days of Dox treatment is indicated on the X axis. Fresh medium or medium with 0.3 μg/ml Dox was supplemented every two days. At the end of time course (day 10), all samples were assessed for viability by CellTiter-Glo (Promega). Values were normalized to untreated samples of each inducible cell line. Three biological replicas were performed. Data are presented as mean (SD).

We observed that MCPyV ST binds specifically to MYCL and the EP400 complex. However, it was not clear if any of these factors were required for proliferation. To identify essential genes in MKL-1 cells, we performed a CRISPR-Cas9 screen of 18,493 genes using two pooled sgRNA libraries H1 and H2, each containing 5 unique sgRNAs for each gene. Using the MAGeCK-VISPR analysis pipeline, Gene Set Enrichment Analysis (GSEA) of known human housekeeping genes revealed that these genes were significantly negatively correlated with the results of the CRISPR screen [31] (S3A Fig). After accounting for copy number variations in MKL-1 cells, we identified 481 genes that were negatively selected in the CRISPR-Cas9 screen with a false discovery rate (FDR) < 0.05 [32], of which 276 have been classified as housekeeping genes (S3B and S3C Fig, S2 Table) [33]. Among the 205 genes not classified as housekeeping genes, 79 genes were identified with FDR < 0.01 and the remaining 126 genes were identified with FDR > 0.01 but < 0.05. MYCL, EP400 and RUVBL2 were identified as essential (FDR < 0.05, Fig 3A). Additional components of the EP400 complex were identified in the CRISPR-Cas9 negative selection screen with p-values < 0.05 but with higher FDR values and included KAT5, TRRAP, DMAP1, ING3 and YEATS4.

Given the requirement of MYCL for viability of MKL-1 cells in the CRISPR-Cas9 screen and the presence of MYCL in the ST-EP400 complex in both MKL-1 and WaGa cell lines (Fig 1B and S1 Table), we examined the levels of the three MYC family members in MCC cell lines. Six different virus-positive MCC cell lines that expressed ST and LT also expressed MYCL while some had low levels of MYCN and none expressed full length MYC (Fig 3B). For controls, we tested HCT116 cells that predominantly expressed MYC and Kelly neuroblastoma cells that expressed MYCN. The virus-negative MCC cell line UISO did not have detectable levels of MYCL until a C-terminal epitope tagged ST (ST-CT) was introduced (Fig 3B).

To determine if MYCL was required for ST to binding to the EP400 complex, we generated MKL-1 cells that contained doxycycline (Dox) inducible shRNA (shMYCL) or miRNA (mirMYCL) that specifically targeted MYCL. Expression of shMYCL or mirMYCL led to reduced levels of MYCL and decreased MAX co-precipitation of EP400, TRRAP, KAT5 and ST (Fig 3C). Notably, depletion of MYCL reduced the ability of ST to co-precipitate the EP400 complex and reduced EP400 binding to ST (Fig 3C).

Omomyc is a modified fragment of MYC that can bind to MAX and disrupt endogenous MYC-MAX heterodimers [34]. To test if MYCL-MAX heterodimers were necessary for ST interaction with the EP400 complex, we introduced a Dox-inducible, HA-tagged, Omomyc construct into MKL-1 cells. When expressed, HA-Omomyc co-precipitated MAX as expected but not MYCL, ST or subunits of the EP400 complex and led to decreased levels of both MAX and MYCL (Fig 3D). However, ST retained the ability to co-precipitate components of the EP400 complex but not MYCL or MAX when Omomyc was expressed, indicating that ST can bind to the EP400 complex independent of the MYCL/MAX heterodimer. The viability of MKL-1 cells was decreased when MYCL levels were depleted by shMYCL or mirMYCL and when the MYCL-MAX heterodimer was disrupted by Omomyc (Fig 3E).

To determine regions of MYCL that contributed to ST and EP400 binding, a series of HA tagged C-terminal constructs of MYCL was stably expressed in HCT116 cells in the presence or absence of ST. When ST was present, MYCL robustly co-precipitated TRRAP, EP400, YEATS4, DMAP1 as well as ST (S3D Fig). In the absence of ST, WT MYCL co-precipitated MAX but only weakly bound to EP400 complex components. In contrast, the N-terminal 165 residues of MYCL, unable to bind MAX, could co-precipitate ST and the EP400 complex in the presence or absence of ST (S3D Fig). The N-terminal 165 residues of MYCL contains several highly conserved MYC homology boxes (MB) that function to bind MYC modifying proteins [35]. MB1 binds to FBXW7 and MB2 contributes to TRRAP binding (S1 Fig) [21, 36]. We generated HCT116 cells that stably expressed HA-tagged MYCL full length constructs with small in-frame deletions of MB1 or MB2. An HA IP for ΔMB2 MYCL co-precipitated ST and MAX but not the EP400 complex, while the ΔMB1 MYCL co-precipitated MAX but neither ST or the EP400 complex (S3D Fig). These data indicate that MB1 and MB2 of MYCL contribute to ST and EP400 complex binding.

### ST binds TRRAP and MYCL in absence of EP400

**Fig 4:**
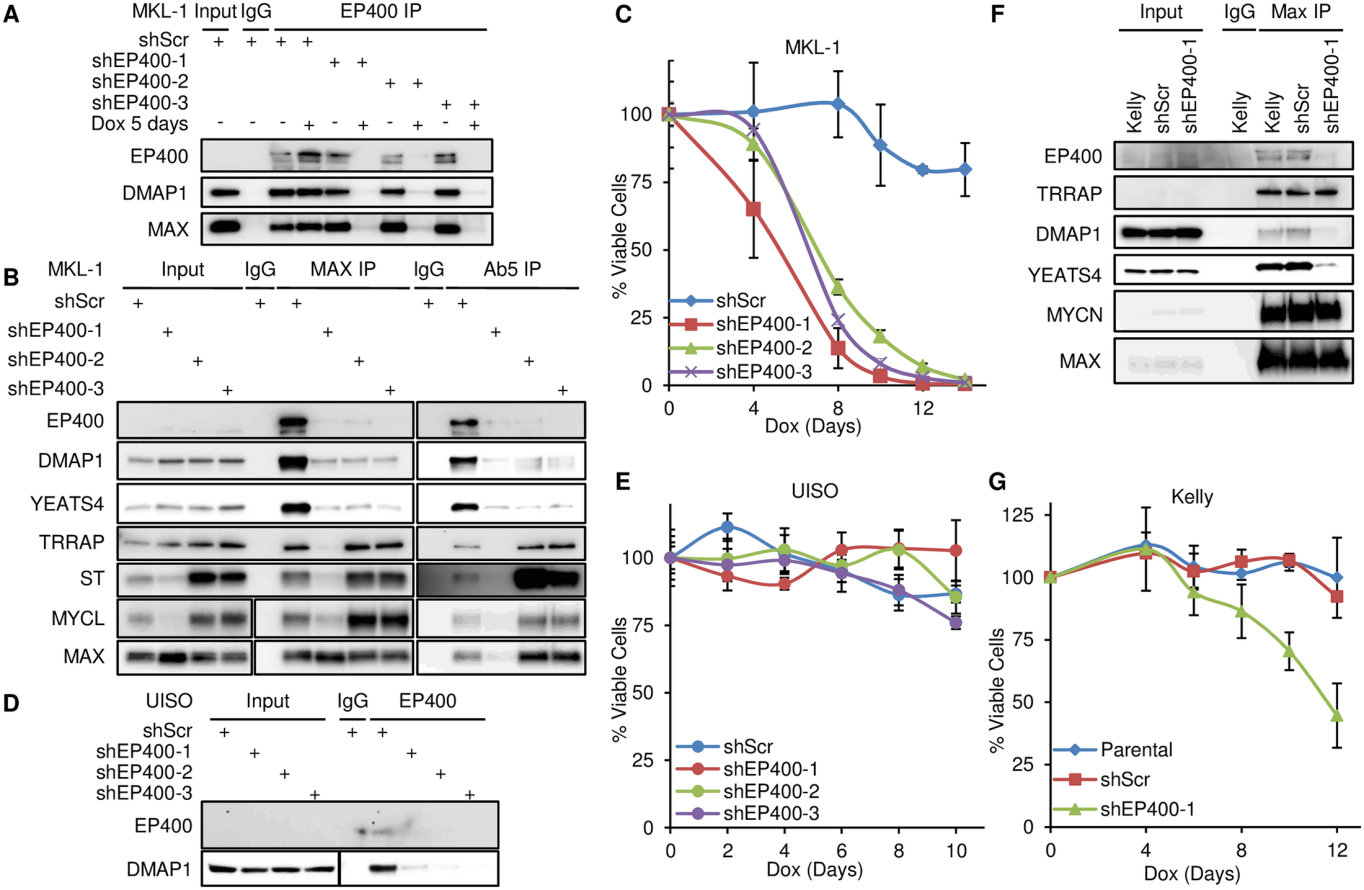
ST binds TRRAP and MYCL in absence of EP400. (A) MKL-1 cells containing three different Dox-inducible shRNA targeting EP400 (shEP400–1, shEP400-2, or shEP400-3) or shScramble (shScr) treated with Dox (0.3 μg/ml) every two days for five days. Lysates (Input) were immunoprecipitated with EP400 or control IgG antibodies and blotted for cells before (-) or after (+) 5 days of Dox treatment. (B) Same as 4A except lysates were immunoprecipitated with control IgG, MAX or Ab5 antibodies and blotted for cells after (+) 5 days of Dox treatment. (C) Cell viability assay of MCPyV positive MCC cell line MKL-1 containing Dox-inducible shRNA targeting EP400 (shEP400) or scramble (shScr). Dox added for indicated number of days. Three biological replicas were performed. Data are presented as mean (SD). (D) Lysates from UISO cells containing an inducible scramble shRNA (shScr) or 3 different shRNAs specific for EP400, prepared after 5 days Dox treatment were immunoblotted (Input) or immunoprecipitated with EP400 antibody or control IgG and blotted with indicated antibodies. (E) Cell viability assay of MCPyV negative MCC cell line UISO containing Dox-inducible shRNA targeting EP400 (shEP400) or scramble (shScr). Dox added for indicated number of days. Three biological replicas were performed; data are presented as mean (SD). (F) Lysates from parental Kelly cells or containing Dox inducible scramble shScr or shEP400-1 prepared after 5 days Dox treatment were immunoblotted (Input) or immunoprecipitated with MAX antibody or non-specific IgG and blotted with antibodies indicated. (G) Cell viability assay of Kelly cells containing Dox-inducible shRNA targeting EP400 (shEP400) or scramble (shScr). Three biological replicas were performed; data are presented as mean (SD).

To test the requirement for EP400 in virus-positive MCC, we generated MKL-1 cell lines containing three different dox-inducible shRNAs targeting EP400 (Fig 4A). In the presence of dox, levels of EP400 were reduced and an immunoprecipitation for EP400 was unable to co-precipitate DMAP1 or MAX (Fig 4A). Of note, knockdown with shEP400-1 led to decreased levels of ST and MYCL in addition to lower levels of EP400 (Fig 4B). In contrast, shEP400-2 and shEP400-3 reduced EP400 levels but did not affect ST and MYCL levels. When EP400 levels were reduced by shEP400-2 or -3, MAX and ST retained the ability to co-precipitate each other, as well as MYCL and TRRAP but not DMAP1 or YEATS 4 (Fig 4B). The viability of MKL-1 cells decreased significantly upon depletion of EP400 with each of the 3-inducible shRNA (Fig 4C).

To determine if the effect of EP400 depletion was specific to virus-positive MCC cell lines, we introduced the 3 inducible shEP400 constructs into the virus-negative MCC line UISO. We confirmed that knockdown of EP400 led to reduced levels of EP400 in UISO cells (Fig 4D). In contrast to the MCPyV-positive MCC cell line MKL-1, the viability of UISO cells was unaffected by EP400 knock-down (Fig 4E). Given that the Kelly neuroblastoma cell line is dependent on continued expression of MYCN, we tested its sensitivity to EP400 depletion [37]. Depletion of EP400 in Kelly cells by shEP400-1 led to reduced binding of MAX to DMAP1 and YEATS4 while retaining binding to MYCN and TRRAP (Fig 4F). Of note, shEP400-1 did not affect MYCN levels in Kelly cells. As shown in Fig 4G, Kelly cells had reduced viability when EP400 levels were reduced.

### MCPyV ST, MYCL and EP400 complex cooperate to reprogram and transform cells

**Fig 5:**
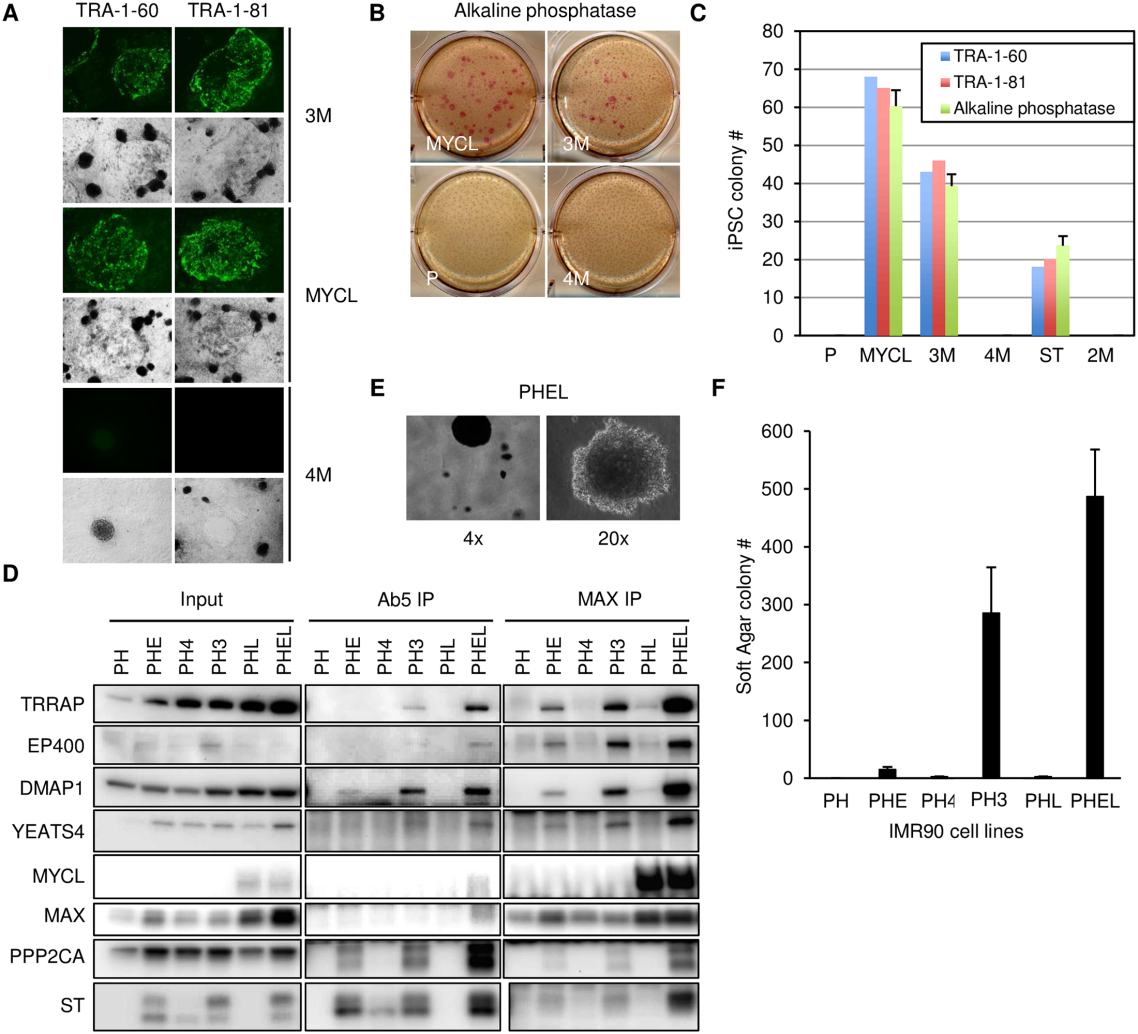
MCPyV ST, MYCL and EP400 complex cooperate to reprogram and transform cells. (A) HFK-hTERT cells were transduced with Dox-inducible OCT4, SOX2 and KLF4 (P) and stably expressed MYCL, 3M or 4M MCPyV ST. Cells were treated with Dox for 31 days and then were immunostained with fluorescent antibodies to TRA-1-60 or TRA-1-81. Light field images demonstrate flat iPSC colonies formed with 3M and MYCL but not from 4M. (B) Cells were stained with alkaline phosphatase one day after immunostaining (Fig 5A). (C) Number of iPSC colonies detected after 31 days. Three biological replicas were performed. Data are presented as mean (SD). (D) IMR90 cells stably expressing dominant negative p53 and hTERT (PH) were transduced with MYCL (PHL) or tumor derived MCPyV ER region containing truncated LT and wild type ST (PHE) and MYCL (PHEL) or 3M mutant ST (PH3) and 4M mutant ST (PH4). Lysates (Input) were prepared from indicated cells, immunoprecipitated with Ab5 or MAX antibodies followed by immunoblotting with the indicated antibodies. (E) Images of soft agar colonies from PHEL cells (4X or 20X magnification). (F) Anchorage independent growth of IMR90 cells indicated in D (10^5^ cells) plated in soft agar and cultured for 4 weeks. Three biological replicas were performed. Data are presented as mean (SD).

Expression of MYC or MYCL together with OCT4, SOX2 and KLF4 (OSK) can generate induced pluripotent stem (iPS) cells from a variety of somatic cell types [38, 39]. Furthermore, MYC interaction with the EP400 complex has been implicated in the generation and maintenance of embryonic stem (ES) and iPS cells [40, 41]. Given that MCPyV ST can bind to MYCL and the EP400 complex, we tested its ability to contribute to iPS cell generation. Since keratinocytes have higher reprogramming efficiency compared to other cell types due to lower p53 and p21 protein levels [42], we generated hTERT-immortalized human keratinocytes with an inducible OSK expression vector and stably introduced MYCL, ST or ST mutants. Expression of OSK in the presence of MYCL, ST or 3M led to the appearance of flat human ES cell-like colonies with defined borders that could be stained by alkaline phosphatase and ES cell surface markers TRA-1-60 and TRA-1-81 (Fig 5A–5C) [43]. In contrast, the ST EP400-binding defective 2M and 4M mutants were unable to generate iPS cells. These results indicate that ST binding to MYCL and the EP400 complex was able to cooperate with OSK to promote the generation of iPS cells.

We examined if ST mediated transformation was dependent on interaction with MYCL and the EP400 complex. When a MCC tumor-derived MCPyV early region (E) that encoded truncated LT and wild type ST was expressed in IMR90 human diploid fibroblasts, we observed a senescent phenotype with elevated levels of p53 and p21 [23]. To suppress this phenotype, we stably expressed a dominant negative form of p53 (p53DD abbreviated as P) and hTERT (H) in IMR90 cells to generate PH cells [44]. The PH cells tolerated MCPyV early region with wild type ST (PHE), 3M ST (PH3) or 4M (PH4) mutant ST, and exogenous MYCL (PHL) without undergoing senescence. Immunoprecipitation of ST with Ab5 from PHE cell lysates revealed a weak interaction with DMAP1, a component of the EP400 complex (Fig 5D). However, when MYCL was co-expressed with wild type ST in PHEL cells, ST and MAX readily co-precipitated the EP400 complex. The 3M ST mutant could efficiently co-precipitate the EP400 complex even without exogenous MYCL expression (PH3). In contrast, the 4M ST mutant (PH4) was unable to co-precipitate the EP400 complex.

We tested the ability of these fibroblasts to grow in an anchorage-independent manner when cultured in soft agar. IMR90 cells expressing p53DD and hTERT (PH) alone or with MYCL (PHL) were unable to form colonies. Cells expressing the MCPyV early region (PHE) formed a few colonies, while co-expression of MYCL (PHEL) led to an increased number of soft agar colonies (Fig 5E and 5F). The highly expressed 3M ST mutant (PH3) could induce anchorage-independent growth while the 4M mutant (PH4) failed to form soft agar colonies. The number of colonies formed for each cell type reflected the relative binding of ST and MAX to the EP400 complex in the presence of the various ST constructs (compare Fig 5D and 5F).

### MAX, EP400 and MCPyV ST bind to actively transcribed promoters

**Fig 6:**
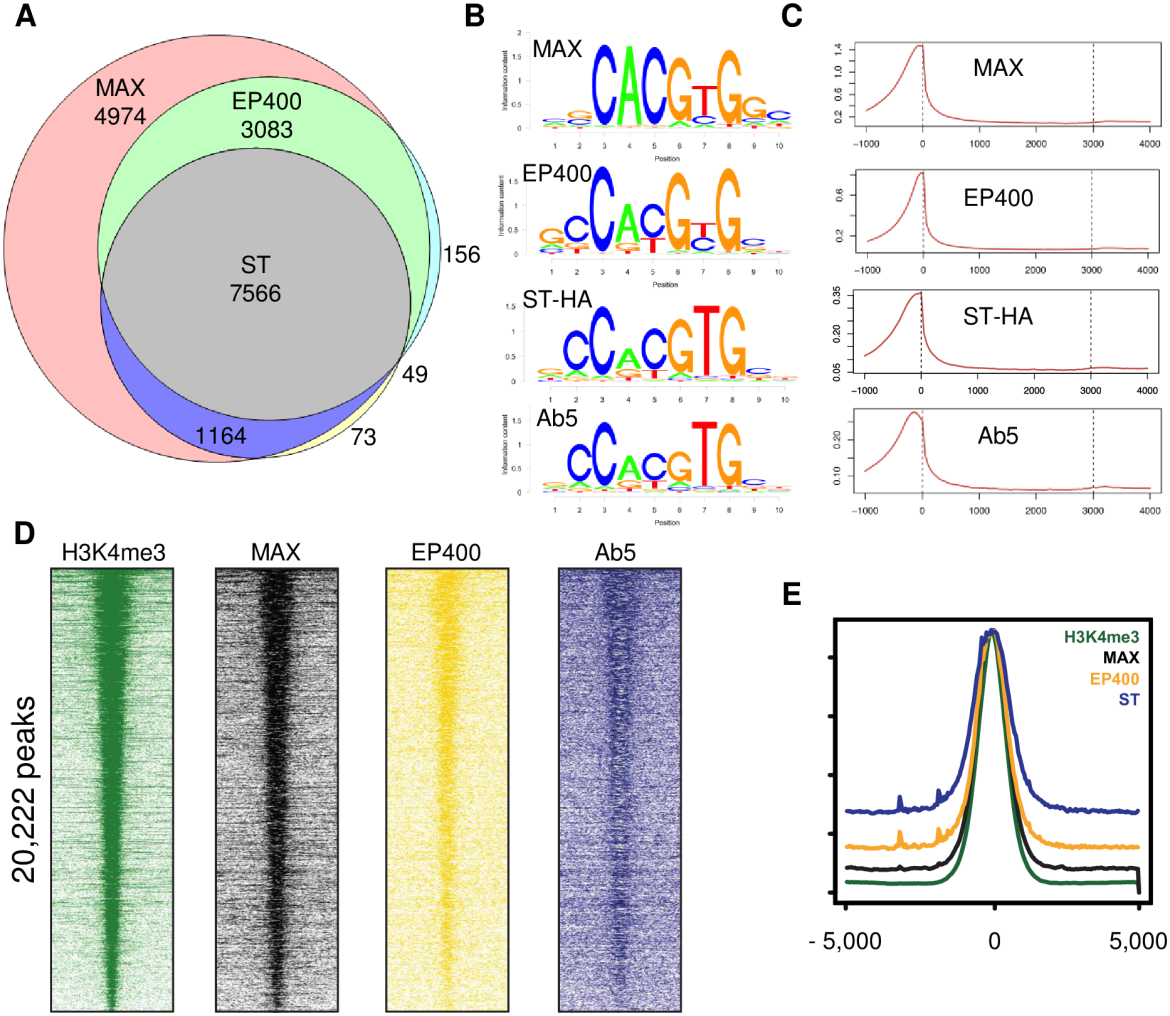
MAX, EP400 and MCPyV ST bind to actively transcribed promoters. (A) Venn diagram of annotated genes corresponding to peaks identified by ChIP-seq with indicated antibodies. Two biological replicas of MAX and EP400 were performed and shared genes indicated. Shared genes identified with Ab5 and ST-HA are indicated. See also S6A Fig. (B) De novo DNA motif identification with indicated antibodies. (C) Distribution of peaks by Metagene analysis. (D) Heatmaps of H3K4me3, MAX, EP400 and ST (Ab5) ChIP peaks ranked by read density of H3K4me3 and scaled against the 75th percentile of genome-wide read density for each ChIP. (E) Meta-track analysis of ChIP-seq read density for MAX, EP400 and ST at all H3K4me3 peaks genome-wide. Regions are centered and ranked for H3K4me3 peaks over input.

Given the known chromatin binding activities of MYCL and the EP400 complex, we tested if MCPyV ST could bind specifically to DNA. We performed chromatin immunoprecipitation followed by sequencing (ChIP-seq) with the mass spectrometry-validated antibodies to EP400 and ST. Since no antibody suitable for immunoprecipitation or ChIP was available for MYCL, we performed ChIP-seq with the MAX antibody. Although it has been reported that MCPyV truncated LT does not bind to chromatin, we considered the possibility that ChIP with Ab5 might also enrich for chromatin-bound LT [6]. To account for this possibility, we generated a MCC cell line (MKL-1) derivative that stably expressed MCPyV ST with a C-terminal HA epitope tag and performed ChIP with an HA antibody. Replicas of MAX and EP400 ChIP-seq identified many peaks that were also identified by anti-ST (Ab5) and anti-HA ChIP-seq. Common gene targets were identified by assigning peaks to the nearest genes (Fig 6A and S4A Fig). *De novo* DNA motif analysis identified the MYC target E-box sequence CACGTG as the most frequently observed motif with Z-scores -42.1726, -20.0773, -23.9634, -19.137 for MAX, EP400, ST-HA and Ab5 antibodies respectively (Fig 6B). Peaks were highly enriched for promoters and 5’UTR sequences (Fig 6C and S4B Fig). ChIP for MAX, EP400 or ST followed by re-ChIP for these three factors indicated that they could bind simultaneously to DNA (S4C Fig).

Given the strong enrichment for promoters, we performed ChIP-seq with antibodies to histone H3 modified by lysine 4 trimethylation (H3K4me3), a histone mark enriched at actively transcribed gene promoters [45]. H3K4me3 ChIP-seq identified 20,222 peaks with MAX, EP400 and ST centered on the same peaks (Fig 6D and 6E). These results indicate that MAX, EP400 and MCPyV ST bind as a complex specifically to E boxes near the transcription start sites (TSS) of actively expressed genes.

We validated the ChIP-seq experiments on several promoters. We prepared chromatin from MKL-1 cells after transduction with vectors expressing shMYCL, mirMYCL or controls and performed ChIP with Ab5. As shown in S5A–S5C Fig. we observed ST binding to the MYCL gene as well as three additional gene promoters that were significantly reduced by MYCL depletion. We also prepared chromatin from MKL-1 cells containing the inducible shEP400-1 before and after dox addition. We observed strong MAX binding to several gene promoters that was reduced upon EP400 depletion (S5D Fig).

### Specific gene regulation by ST-MYCL-EP400

**Fig 7:**
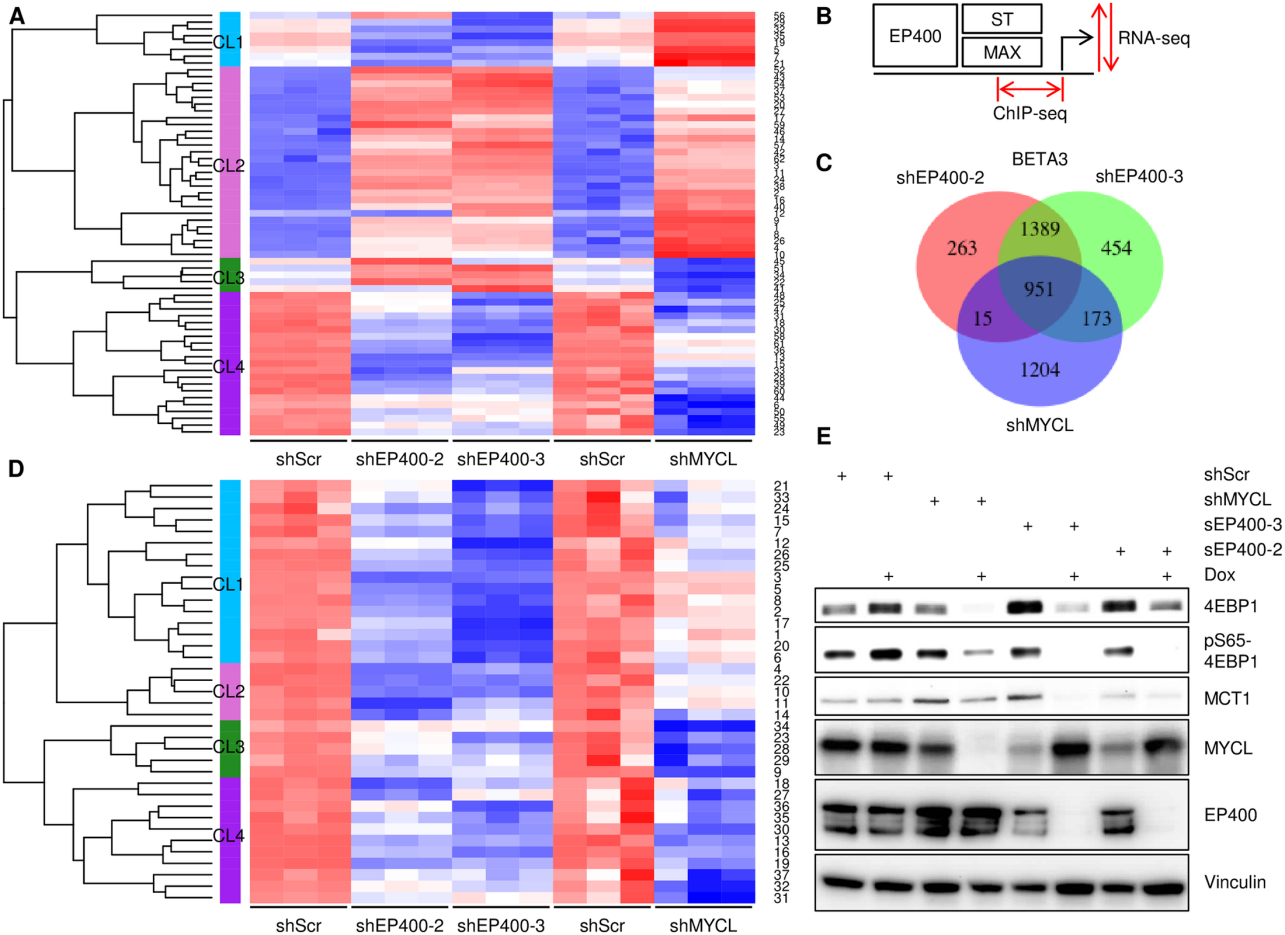
MCPyV ST cooperates with MYCL and EP400 complex to activate gene expression. (A) Heatmap depicts average mean-centered and standard-deviation-scaled gene expression profiles for each of 62 clusters created by applying model-based clustering to the differentially expressed genes (DEG) in MKL-1 cells after depletion of EP400 or MYCL in comparison to shScr control. Model-based clusters (1–62) are labeled on the right-hand side and their gene members are listed in S3 Table. Merged Clusters (CL1-4) are indicated on the left-hand side. (B) Diagram illustrating BETA Activating/Repressing Function Prediction of transcription factors by correlation of distance of peaks from corresponding TSS obtained in ChIP-seq of ST, MAX and EP400 with changes in gene expression by RNA-seq after Dox-induction with shRNA targeting EP400 or MYCL. (C) Venn diagram showing common direct target genes of MAX, EP400 and ST identified by BETA based on ChIP-seq of MAX, EP400, ST and RNA-seq of shEP400–2, -3 and MYCL shRNA (BETA3). (D) Heatmap depicts average mean-centered and standard-deviation-scaled gene expression profiles for each of 37 clusters created by applying model-based clustering to the 951 BETA3 target genes in MKL-1 cells after depletion of EP400 or MYCL in comparison to shScr control. Model-based clusters (1–37) are labeled on the right-hand side and their gene members are listed in S3 Table. Merged Clusters (CL1-4) are indicated on the left-hand side. (E) MKL-1 cells containing Dox inducible shRNA for shScr, shMYCL or EP400 (shEP400-2, -3) were treated with dox for 5 days. Lysates were blotted with indicated antibodies. EP400 immunoprecipitations were blotted with EP400 antibody.

To identify genes and associated biological functions that are controlled by the ST-MYCL-EP400 complex, we performed RNA-seq of MKL-1 cells containing inducible shMYCL, shEP400-2, shEP400-3 and shScr with RNA isolated from cells treated with dox for 5 days. The differentially expressed genes (DEG) list consists of 2157 genes that passed the cutoff *P*_adj_ < 0.001 in all three comparisons (shEP400-2, shEP400-3 and shMYCL vector, relative to shScr control). To create heatmaps, counts were normalized separately for the two experiments (shEP400 and shMYCL) and then corrected for batch effect using ComBat (S6 Fig) [46]. These genes were first grouped into 62 clusters using model-based clustering [47]. The average expression profiles of each cluster were then merged into four general patterns of behavior using hierarchical clustering (Fig 7A and S3 Table; see Methods for more details). The genes in each of the four merged clusters were evaluated for statistical enrichment in Gene Ontology (GO) biological process terms. Cluster membership and all results of the GO term analysis are presented in S3 Table. We observed that genes upregulated by shEP400 and shMYCL fell into the cluster DEG-CL2 and were enriched in neurogenesis, skin development and hair cycle. DEG-CL4 contained genes downregulated by EP400 and MYCL and were enriched in cellular component biogenesis, RNA processing and amide biosynthetic process. Two smaller clusters represent genes that behaved differently under shEP400 and shMYCL conditions. DEG-CL1 genes were decreased by shEP400, increased by shMYCL and enriched for actin cytoskeleton and regulation of signaling. DEG-CL3 exhibited the opposite pattern of expression and was enriched in nerve development and liposaccharide biosynthesis. These results show that both MYCL and EP400 support cell growth by upregulating bulk synthesis of biomolecules including ribosomes and proteins while simultaneously repressing cell adhesion and developmental programs in neurogenesis and skin.

To integrate expression profiling with the aforementioned ChIP-seq experiments (Fig 6), we performed Binding and Expression Target Analysis (BETA) that links the proximity of the ChIP-seq binding peaks to the TSS with expression level changes in the corresponding genes to predict activating and repressive activities of transcription factors [48] (Fig 7B). We performed BETA analysis for MAX, EP400 and ST ChIP-seq studies with RNA-seq analysis for shEP400-2, shEP400-3 and shMYCL. We observed that the genes whose levels decreased (downregulate) upon EP400 or MYCL depletion were significantly enriched for MAX, EP400 and ST chromatin binding (S7A Fig, S4 Table). In contrast, genes whose levels increased (upregulate) with EP400 depletion were not significantly associated with the MAX, EP400 and ST ChIP-peaks. This indicates that the ST, MYCL/MAX and EP400 complex binding contributes to specific gene activation. We compared the target genes identified for each ChIP-seq analysis with the RNA-seq analysis for shEP400-2, shEP400-3 and shMYCL and identified 951 shared target genes of MAX, EP400 and ST whose levels went down upon EP400 or MYCL depletion and had significant evidence for direct ChIP binding by BETA analysis (BETA3, Fig 7C & S7B Fig). When the RNA-seq data for shEP400-1 was also included in the analysis, a total of 379 target genes were identified (BETA4, S7A–S7C Fig).

We then examined 951 genes identified in BETA3 that were downregulated by shEP400 and shMYCL with evidence of direct binding according to BETA analysis of the ChIP-seq data. We note that these genes exhibited a wide range of fold changes upon depletion of EP400 and MYCL, with 136 of the 951 genes showing greater than 2-fold downregulation due to shEP400 (shEP400 inverse fold change), and 62 out of 951 genes showing greater than 2-fold downregulation due to shMYCL (S8 Fig and S5 Table). To find global patterns of expression that reflected functional regulation, we centered and scaled their expression profiles and created model-based clusters and merged clusters, using the same procedure as in the analysis of the DEG list. The final merged clusters were then evaluated for GO term enrichment. Cluster membership of the BETA3 genes and full GO term enrichment results are listed in S3 Table. We found that these genes naturally divide into two groups: genes that were more strongly affected by shEP400 (BETA3-CL1 and 2) and genes that were more strongly affected by shMYCL (BETA3-CL3 and 4) (Fig 7D). The shEP400 clusters are enriched for nucleobase-containing compound metabolic process and translation initiation and elongation whereas the shMYCL clusters are involved in RNA processing and peptide metabolic processes.

Among the target genes identified in the shEP400-2, -3 and shMYCL depletion analyses was the translational control factor 4EBP1 that has been reported to be upregulated by ST [13]. To test this effect, lysates were generated from MKL-1 cells before or after depletion of EP400 and MYCL were blotted for 4EBP1. As expected, levels of MYCL were depleted by shMYCL and EP400 by shEP400-2 and -3 (Fig 7E). Of note, levels of 4EBP1 and the phosphorylated serine residue 65 form (pS65-4EBP1) were reduced upon EP400 or MYCL knockdown. In addition, we have recently reported that levels of the lactate transporter MCT1 (SLC16A1) increase upon expression of ST [16]. Levels of MCT1 were also decreased upon depletion of EP400 or MYCL (Fig 7E).

We compared the effect of depleting EP400 or MYCL in the virus-positive MKL-1 cells to the effect of expressing ST in normal cells. We examined RNA-seq profiles from IMR90 human fibroblasts with inducible expression of GFP or MCPyV ST over the course of 4 days [16]. As shown in the heatmap in S9 Fig, the genes that were downregulated by shEP400 and shMYCL in MKL-1 cells tend to be upregulated by ST in IMR90 cells consistent with the model that ST activates functional interactions with EP400 and MYCL and their transcriptional targets.

## Discussion

Here, we demonstrate that MCPyV ST specifically recruits the MYCL and MAX heterodimer to the 15-component EP400 complex. These interactions are essential for the transforming function of MCPyV ST, the viability of virus-positive MCC cells and likely to be a major contributor to the oncogenic potential of MCPyV in MCC. Consistent with this model, a genome-wide CRISPR-Cas9 screen revealed that MYCL and several components of the EP400 complex were essential for viability of the virus-positive MCC cell line MKL-1. The interaction of MCPyV ST with MYCL and the EP400 complex is unique to the family of polyomaviruses. To date, no other polyomavirus ST has been reported to bind the EP400 complex or a MYC homolog [15]. Several other viruses have been reported to engage the EP400 complex. Perhaps most similar to the results reported here, the adenovirus E1A oncoprotein binding to MYC and the EP400 complex contributes to its transforming potential [49, 50].

We observed a striking relationship between MYCL and MCPyV ST. MYCL was expressed in all 6 virus-positive MCC cell lines tested. Furthermore, ST appeared to regulate MYCL levels. For example, introduction of ST into several naïve cell lines led to increased levels of MYCL. Conversely, depletion of ST from MKL-1 cells led to decreased levels of MYCL. ST together with EP400 and MAX could bind to the MYCL promoter. In addition, the virus-positive MKL-1 cell line was sensitive to Omomyc expression indicating that the MYCL-MAX heterodimer was required for viability as well as ST interaction. These results are consistent with a positive feedback loop where ST binding to the MYCL promoter contributes to transcriptional activation of MYCL leading to increased levels of MYCL that in turn binds to ST and the EP400 complex.

MYCL has been the forgotten MYC species, since it is not required for normal mouse development and expression is highly restricted in adult tissues [51]. Despite its relative obscurity, MYCL can function as a bona fide oncogene [52, 53]. For example, amplification of MYCL or the presence of the *RLF*-*MYCL1* fusion gene are mutually exclusive of amplifications of MYC or MYCN in small cell lung cancer. Better still, amplification of the 1p34 locus containing the MYCL gene has been reported in MCC suggesting that Merkel cell carcinogenesis is dependent upon excessive MYCL function [54]. Consistent with this report, we estimate that the copy number of MYCL was 3.5 copies in MKL-1 cells based on analyzing ChIP-seq input DNA (S3B Fig, S2 Table). In keeping with this notion, MCPyV ST shows a strong preference for recruiting MYCL to the EP400 complex. It is possible that, despite the widespread presence of MCPyV in healthy individuals, only cells capable of expressing MYCL are susceptible to the oncogenic potential of MCPyV [55, 56].

MYC and MYCL can cooperate with the OSK reprogramming factors to induce a pluripotent state in somatic cells [39, 57]. Comparison of the contributions of MYC to transformation and iPS cell generation show significant overlap with the interaction with the EP400 complex as a key component [41]. Here we find that MCPyV ST can substitute for MYCL in iPS cell generation and that this activity is strictly dependent upon ST interaction with the EP400 complex. Our data indicate that, at least in part, MCPyV ST functions similarly to MYC by binding to the EP400 complex, recruiting it to specific promoters to transactivate gene expression and thereby promoting the generation of iPS cells. These functions may also prove to be critical in establishing and maintaining the oncogenic state of MCC.

Our data reveal that the ST-MYCL-EP400 complex functions, at least in part, to activate specific gene expression. Depletion of MYCL and EP400 led to significant changes in gene expression and cell viability. Those genes whose levels decreased upon MYCL and EP400 depletion were significantly associated with ST, MAX and EP400 binding to their promoters and include classic MYC targets involved in RNA processing, ribosome biogenesis, nitrogen compound and peptide metabolic processes. Additional target genes are involved in cell morphogenesis and signaling in the TNF, WNT, NFκB and DNA damage pathways. Importantly, a large number of metabolic genes were activated by the ST-MYCL-EP4000 complex including a number of transporters including SLC16A1 and SLC7A5 and the MYC-metabolism genes MLX and MLXIP (Mondo) [16, 58]. Factors that promote transcription elongation were also highly enriched including EIF4E, EIF4EBP1, EIF5A [13].

Interestingly, genes whose levels increased upon MYCL or EP400 depletion were involved in neurogenesis, axon guidance, wound healing and cell-cell adhesion. These results can be interpreted to indicate that ST-MYCL-EP400 complex serves to repress differentiation markers and induce a more primitive, progenitor or embryonic state, consistent with its ability to generate iPS cells.

The MYC family functions to activate gene expression at least in part by interaction with a variety of chromatin factors. In addition to the EP400 complex, MYC can bind to the TRRAP-containing STAGA (SPT3-TAF9-GCN5 acetylase) complex that in turn interacts with Mediator [59]. MYC binds to BRD4 and the pTEFb complex to facilitate transcriptional elongation by release of paused RNA polymerase II [60, 61]. The conserved Myc Boxes contribute to transformation with the Myc Box 3b (MB3b) binding to WDR5 and Myc Box 4 (MB4) binding to HFCF1 (S1 Fig) [62, 63]. MB3a, or simply referred to as MB3, found only in MYC and MYCN and not MYCL, is required for tumorigenic activity of MYC in vitro and in vivo [64] and contributes to transcriptional repression by recruiting HDAC3 [65]. At oncogenic expression levels, MYC interacts with MIZ-1 (ZBTB17) to repress transcription, which can be disrupted by mutating valine 394 (V394) in the helix-loop-helix (HLH) domain [66]. We only detected the EP400 complex and did not detect any of these other MYC binding factors in any of the ST complexes. Both MB1 and MB2 of MYCL contribute to ST and MYCL binding. Of note, it appears that ST and MYCL bind directly to TRRAP as evidenced by co-precipitation of TRRAP with ST and MAX antibodies after EP400 depletion (Fig 4B).

In contrast to MCPyV ST, transformation by SV40 ST is strictly dependent on its interaction with PP2A. SV40 ST binding to PP2A perturbs its ability to de-phosphorylate certain substrates including MYC that in turn leads to higher levels of MYC. While it has been reported that MCPyV ST interaction with PP2A is not required for its transforming function, it is possible that PP2A contributes to activity of the MYCL-EP400 complex.

These results highlight an important mechanism for MCPyV ST mediated transformation. The ST-MYCL-EP400 complex functions as a powerful engine to transactivate gene expression and promote oncogenesis. Important questions to be pursued include whether any of the specific downstream transcriptional targets of the ST-MYCL-EP400 complex contribute to MCC oncogenesis and if any of these target genes provide a therapeutic opportunity for virus-positive MCC. In addition, the ST-MYCL-EP400 complex itself may provide a therapeutic opportunity to disrupt interactions between ST, MYCL and the EP400 complex or with any of its components to DNA. In addition, these results may help to explain why the oncogenic activity of MCPyV ST is limited to MCC because of its dependency on MYCL or if this virus is capable of inducing MYCL and cancers in other tissue types.

## Methods

### Ethics statement

Human Subject Research performed in this study complied with all relevant federal guidelines and institutional policies. The Dana-Farber Cancer Institute Institutional Review Board (IRB) approved the study. All subjects were adults and provided informed written consent.

### Cells

MCC cell lines MKL-1, MKL-2 and MS-1 were gifts from Masa Shuda (University of Pittsburgh, PA); MCC cell lines WaGa and UISO from Jürgen Becker (Medical University Graz, Austria); MCC cell lines PeTa and BroLi from Roland Houben (University of Wuerzburg, Germany). Kelly neuroblastoma cell line was a gift from Rani George (Dana-Farber Cancer Institute, MA). 293T, HCT116 and IMR90 cells were obtained from ATCC. HFK-hTERT cells were a gift from Karl Münger (Tufts University, MA).

### DNA

MCPyV early region was PCR amplified from DNA extracted from a Merkel cell carcinoma sample [23]. The cDNA for ST was modified to eliminate the LT splice donor by introducing silent mutations (GAG|GTCAGT to GAa|GTCtcc). Additional ST mutants were generated using QuikChange Lightning Site-Directed Mutagenesis Kit (Agilent).

The EP400, MYCL shRNA target sequence was designed using Block-iT RNAi Designer (Life Technologies) and annealed forward and reverse oligos of hairpin sequence were cloned between AgeI/EcoRI sites of the doxycycline inducible shRNA vector Tet-pLKO-puro (a gift from Dmitri Wiederschain, Addgene #21915) [67]. The MYCL miRNA target sequence was designed using Block-iT RNAi Designer and cloned into pcDNA 6.2-GW/EmGFP-miR vector (Life Technologies) and the pre-miRNA expression cassette targeting MYCL was transferred to pLIX_402 Dox-inducible expression vector via consecutive BP and LR recombination reactions to generate pLIX-mirMYCL plasmid. shRNAs constitutively expressed from lentiviral PLKO vector targeting MCPyV LT/ST (shPanT), ST (shST) or scramble (shScr) have been published before [13, 30, 68].

pMXs-Hu-L-Myc was a gift from Shinya Yamanaka (Addgene # 26022) [39]. MYCL was PCR amplified with C-terminal 3xHA tag or with original stop codon and cloned into pLenti-CMV gateway vector. Omomyc was a kind gift from Sergio Nasi (Sapienza University of Rome, Italy), modified by PCR amplification to include C-terminal HA tag and cloned into pLIX_402. The OCT4-2A-SOX2-2A-KLF4 polycistronic coding sequence was PCR amplified from pKP332 Lenti-OSK1 (Addgene #21627) [69] and cloned into pLIX_402.

Expression vectors include pLenti-CMV (a gift from Eric Campeau, Addgene #17451) [70], doxycycline inducible lentiviral gateway expression vector pLIX_402 (a gift from David Root, Addgene #41394). Lentiviral packaging plasmid psPAX2 and envelope plasmid pMD2.G were gifts from Didier Trono (Addgene #12260, #12259). Retroviral packaging plasmid pUMVC3 was a gift from Robert Weinberg (Addgene # 8449) [71] and envelope plasmid pHCMV-AmphoEnv from Miguel Sena-Esteves (Addgene # 15799) [72]. Retroviral plasmids pBabe-neo-p53DD and pBabe-hygro-hTERT were previously described [44].

Packaging and envelope plasmids were co-transfected with lentiviral or retroviral expression vectors into 293T cells using Lipofectamine 2000 (Life Technologies). Two days after transfection, 293T cell supernatant was purified with 0.45 μm filter and supplemented with 4 μg/ml polybrene before transducing recipient cells. Stable cell lines were generated after selection with 1–2 μg/ml puromycin, 5–10 μg/ml blasticidin, 500 μg/mL neomycin, and 100 μg/mL hygromycin as required by each vector.

### Cell viability assay

CellTiter-Glo Luminescent Cell Viability Assay was performed according to the protocol from Promega. Basically, 3000 MKL-1 parental or dox-inducible cells were plated in 96 well plate. Fresh medium was supplemented every two days with or without doxycycline. The number of days that cells had been treated with doxycycline was labelled on X-axis. At the end of time course, CellTiter-Glo reagents were added to lyse cells. For each cell line, doxycycline treated samples were normalized to untreated samples.

### Anchorage independent growth assay

Anchorage independent growth was performed as described [23] using 6-well dishes with SeaPlaque Agarose (Lonza) at concentrations of 0.3% top and 0.6% bottom layers. Agarose was diluted with 2X MEM (Gibco) supplemented with 2X Gluta-max (Gibco), 2X pen-strep (Gibco), and 30% FBS. IMR90 cells (10^5^) were seeded in triplicate in the top agarose layer. Wells were fed with top agarose twice per week. After 4 weeks, cells were stained with 0.005% crystal violet (Sigma) in PBS and colonies were counted. Statistical significance was determined by ordinary one-way ANOVA for multiple comparisons with p < 0.05.

### iPS cell generation

HFK-hTERT cells were transduced with pLIX-OSK and selected with puromycin to establish the parental cell line (P) followed by transduction with MYCL or ST in pLenti-CMV vector and selection with blasticidin. 200,000 cells were seeded in Matrigel (BD Biosciences) coated 6-well plate in triplicate on day 0 in Keratinocyte-SFM medium (Gibco) supplemented with 0.5 μg/ml doxycycline. On day 3, medium was changed to mTeSR1 (Stemcell Technologies) supplemented with doxycycline. iPS colonies were visible under microscope after 3 weeks and stained with StainAlive TRA-1-60 or TRA-1-81 antibodies (Stemgent) and Alkaline Phosphatase Detection Kit (Millipore).

### Immunoprecipitation and immunoblotting

The following antibodies were used: Ab5 and Ab3 [23, 73]; HA (Abcam); EP400, RUVBL2 (Bethyl); MAX, KAT5, DMAP1, MNT (Santa Cruz); MYCL (R&D Systems); ING3 (Sigma); PPP2CA (BD Biosciences); and H3K4me3 (Millipore; 07–473).

Cell lysates were prepared in EBC Lysis buffer (50 mM Tris pH 8.0, 150 mM NaCl, 0.5% NP-40, 0.5 mM EDTA, 1 mM β-Mercaptoethanol and freshly added protease inhibitor and phosphatase inhibitor cocktail). Immunoprecipitations were performed with protein G Dynabeads (Life Technologies) mixed with immunoprecipitation antibodies or anti-HA magnetic beads (Pierce Biotechnology). After overnight incubation on a rotating apparatus at 4°C, magnetic beads were washed with high salt wash buffer (50 mM Tris pH 7.4, 300 mM NaCl, 0.5% NP-40, 0.5 mM EDTA) five times. Bound proteins were eluted from magnetic beads with 2x Laemmli sample buffer (Bio-Rad). After electrophoresis, the separated proteins were transferred to PVDF membrane and blotted. Immunoblots were developed using Clarity Western ECL substrate (Bio-Rad) and imaged with G:BOX Chemi system (Syngene).

### MudPIT

MudPIT was performed with MKL-1 or WaGa suspension cells (30 x 15-cm diameter plates) harvested in 30 ml EBC lysis buffer. Clarified cell extract (100–300 mg) was incubated overnight at 4°C with 30 μg antibodies crosslinked to 30 mg protein G agarose beads by dimethyl pimelimidate (DMP). Beads were washed with high salt wash buffer five times, then eluted with 0.2 M glycine pH 3 and neutralized with 1 M Tris pH 8.0. Proteins were precipitated with 1/5 TCA overnight at 4°C and washed with cold acetone twice and analyzed by MudPIT as described [74]. The triple-phase fused-silica microcapillary column was packed with 8–9 cm of 5-μm C18 Reverse Phase (Aqua, Phenomenex), followed by 3 to 4 cm of 5-μm Strong Cation Exchange material (Partisphere SCX, Whatman) and 2 to 3 cm of C18 RP and equilibrated with Buffer A (5% ACN, 0.1% Formic Acid). A10-step chromatography run was performed with the last two chromatography steps consisting of a high salt wash with 100% Buffer C (500mM Ammonium Acetate, 5% ACN, 0.1% Formic Acid) followed by an acetonitrile gradient to 100% Buffer B (80% ACN, 0.1% Formic Acid). 2.5 kV voltage was applied distally to electrospray the eluting peptides. Full MS spectra were recorded on the peptides over a 400 to 1,600 m/z range, followed by five tandem mass (MS/MS) events sequentially generated in a data-dependent manner on the first to fifth most intense ions selected from the full MS spectrum (at 35% collision energy).

### Gel filtration

A frozen pellet of MKL-1 cells was resuspended in mammalian cell lysis buffer (MCLB; 50mM Tris pH 7.8, 150 mM NaCl, 0.5% NP40) in the presence of protease and phosphatase inhibitors (Roche Complete, EDTA-free Protease Inhibitor Cocktail and 25 mM sodium fluoride, 1 mM sodium orthovanadate, 5 mM β-glycerophosphate). The lysate was incubated on ice for 15 minutes then clarified by centrifugation in a refrigerated microfuge for 10 minutes at top speed. The supernatant was further clarified using 0.45 μM Durapore PVDF spin filters (Millipore). Approximately 7 mg of total cellular protein was applied to a Superose 6 10/300 GL column run in an AKTA pure FPLC (GE Healthcare) with MCLB as the running buffer. The injection volume was 500 μl, the flow rate was 0.5 ml/minute, and 0.5 mL fractions were collected from 0.2 column volumes to 1.5 column volumes. The molecular weights were estimated by loading 1 mg of individual protein standards from the Gel Filtration Markers Kit for Protein Molecular Weights 29,000–700,000 Da (Sigma-Aldrich).

### RNAi knockdown of MCV T antigens

MCV T antigens were knocked down in MKL-1 cells using shRNAs previously published [13, 30]. The shRNAs were cloned into pLKO.Puro vectors, lentivirus was generated in 293T cells using psPax2 and pVSV.G vectors, and MKL-1 cells were infected using spinoculation (centrifugation at 800g for 30 mins with viral supernatants) followed by infection overnight in the presence of 1 μg/ml Polybrene. 24 hours post infection, MKL1 cells were spun down and resuspended in medium containing puromycin (1 μg/ml). Cells were harvested after 72 hours and processed for immunoblotting and immunoprecipitation.

### Genome-wide CRISPR screen

CRISPR lentiviral libraries H1 and H2 each contain 92,817 pooled sgRNAs targeting 18,493 human genes. CRISPR screen was performed by following a previous protocol (https://doi.org/10.1101/106534). Briefly, 2x10^8^ MKL-1 cells were transduced with H1 and H2 CRISPR libraries separately at MOI 0.3 to ensure single sgRNA incorporation per cell. After 6 days of 1 μg/ml puromycin selection, surviving cells from each sgRNA library transduction were split in half, 3x10^7^ cells were saved as initial state controls, the rest were cultured for a month with at least 3x10^7^ cells maintained and used as final state samples. Genomic DNA was extracted and 200 μg from each sample were used to PCR amplify integrated sgRNAs and to generate 4 libraries for next generation sequencing. 50 million reads were obtained for each sequencing library. To filter out false positive targets due to strong correlation between decreased cell viability and increased gene copy number in CRISPR/cas9 screens [32], copy numbers of every 50-kb segment of MKL-1 genome were called from the input of ChIP-seq experiments using QDNAseq software. Segmented copy numbers were converted to copy numbers per gene based on gene coordinates. MAGeCK-VISPR pipeline was used to assess data quality, correct copy number variation effect and identify statistically significant targets [75].

### ChIP-seq

MKL-1 cells or a derivative stably expressing MCPyV ST with a C-terminal 3xHA tag were used for ChIP. For MAX, EP400, Ab5 and HA antibodies, ChIP was performed as described [76] with the modification that cells were dual cross-linked with 2 mM disuccinimidyl glutarate (DSG) and 1% formaldehyde [77] and sonicated at 4°C with a Branson Sonifier 250 at 20% duty cycle for 1 minute with 1 minute rest in between for 15 cycles. ChIP- reChIP was performed using the Re-ChIP-IT kit (Active Motif). For ChIP-seq, 10 ng of DNA from ChIP experiments or input DNA were prepared for sequencing with NEBNext ChIP-seq Library Prep Reagent Set for Illumina (New England BioLabs). Amplified libraries were cleaned up using AMPure XP beads (Beckman Coulter) and checked on a Bioanalyzer (Agilent) to confirm a narrow distribution with a peak size around 275 bp. Diluted libraries were used for 50 cycles single-end sequencing on HiSeq 2000 system (Illumina) at the Center for Cancer Computational Biology (CCCB) at Dana-Farber Cancer Institute following the manufacturer’s protocol.

H3K4me3 ChIP-seq was performed as described with minor changes [78]. 0.5xE06 MKL-1 cells were cross-linked with 1.1% formaldehyde and sonicated at 4°C with a Bioruptor (Diagenode). Samples were sonicated on the high setting for 30 seconds with 30 seconds rest in between. Libraries for Illumina sequencing were prepared using the ThruPlex FD DNA-seq kit (Rubicon Genomics). Amplified libraries size-selected using a 2% gel cassette in the Pippin Prep system (Sage Science) to capture fragments between 200–700 basepairs. Libraries were run in Illumina Nextseq.

ChIP-seq mapping was performed using Bowtie (version 0.12.7) against human genome version hg19 allowing only uniquely mapping reads. Peak calling was done using MACS2 (version 2.1.0.20140616) on either single replicate mapped files or replicates merged as mapped bam files using the samtools (version 0.1.18-dev (r982:313)) merge function. Top ranking peaks (5000 most significant, p-val as reported by MACS2) and the Macs2 generated tag pileup output was visualized using the Meta Gene signal distribution function of the CEAS (version 0.9.9.7) analysis package [79].

Significantly enriched transcription factor binding element motifs where found using the Cistrome SeqPos tool using the 1000 most significant (Macs2 p-value) peaks [80].

To calculate genome-wide overlap, all enriched H3K4me3 peaks were extended 5kb in each direction, divided into 250 bins and the read density was calculated in each bin. Density was normalized to the largest value observed in each experiment genome-wide and plotted either as an average of all regions (meta plot) or as a heat map.

### RNA-seq

MKL-1 cells containing tet-PLKO-shEP400, tet-PLKO-shMYCL and tet-PLKO-shScramble were used to perform RNA-seq. Cells (10^7^) were collected before and 5 days after dox addition. Total RNA was purified using RNeasy Plus Mini Kit (Qiagen). mRNA was isolated with NEBNext Poly(A) mRNA Magnetic Isolation Module (New England BioLabs). Sequencing libraries were prepared with NEBNext mRNA library Prep Master Mix Set for Illumina (New England BioLabs) and passed Qubit, Bioanalyzer and qPCR QC analyses. 50 cycles single-end sequencing was performed on HiSeq 2000 system.

Reads were mapped to the Hg19 genome by TOPHAT. HTSeq was used to create a count file containing gene names [81]. The R package DESeq2 was used to normalize counts and calculate total reads per million (TPM), and determine differential gene expression. QC was performed to generate a MA plot to display differentially expressed genes.

To create the heatmaps, counts were normalized separately for the two experiments (shEP400 and shMYCL) using “voom” from the R/Bioconductor package *limma*. Data were then corrected for batch effect using ComBat from the R/Bioconductor package *sva*. In ComBat, the normalized gene expression data were fit to a linear model capturing the effects of basal expression, sample conditions, batch variation, and noise [46]. In our case, the sample conditions corresponded to shScr, shEP400, and shMYCL, and the batch variable corresponded to the experiment in which the data were measured. In the final step of ComBat, the best-fit parameters from the linear model were used to subtract only the effect of the batch variable from the data. Principal Components Analysis (PCA) plots of the data before and after ComBat show that shScr samples from the two different batches cluster together after ComBat (S7 Fig). We then subsetted the batch-adjusted data using the “BETA3” list, defined as previously described in the section on BETA analysis of the ChIP-Seq data, or a “DEG” list of genes that were differentially expressed with *P*_adj_ < 0.001 in all three comparisons: shP400-2 vs. shScramble, shEP400-3 vs. shScramble, and shMYCL vs. shScramble. Differential expression was determined using DESeq2. Note that the DEG list includes both up- and down-regulated genes, whereas the BETA3 list includes only down-regulated genes.

For each gene list, the batch-adjusted expression values were first standardized across all 15 samples by mean-centering and scaling so that standard deviations are all set to 1. Genes were then clustered using model-based clustering as implemented in the R package *mclust*. An average profile was created for each gene cluster by taking the mean over the standardized expression values for all the genes in the cluster. Next, the average profiles were merged using complete linkage hierarchical clustering with a Euclidean distance metric. By cutting the tree at a height of 3.5 (for the BETA3 list) or 5 (for the DEG list), we merged the model-based clusters into larger patterns of gene expression. Gene Ontology (GO) term enrichment was run on the final merged clusters using the R/Bioconductor package *GOstats* with the following parameters: the background set consisted of all the genes from the original RNA-seq alignment, the Benjamini-Hochberg method was applied for multiple testing correction, and the conditional hypergeometric test was used to take into account relationships between GO terms. Heatmaps depict the average standardized expression profiles and were created using the “heatmap.2” function from the R package *gplots*.

The IMR90 ST and GFP RNA-seq data is available from the Gene Expression Omnibus (GEO) with accession number GSE79968. The IMR90 data were processed using Tophat and Bowtie, and the log-transformed FPKM values were used for all analysis, as described [16]. The genes in the DEG list that also had non-zero expression values across all IMR90 expression profiles were used to create the final heatmap. To visualize both datasets in the same setting, the IMR90 profiles were each subtracted by a corresponding control, which was defined as the average expression level in the IMR90 GFP cell line at the same time point. The MKL-1 shEP400 profiles were subtracted by the average expression level in the shScr samples from the shEP400 batch. Likewise, the shMYCL profiles were subtracted by the average expression level in the shScr samples from the shMYCL batch. Finally, for each gene, all its expression values across both IMR90 and MKL-1 datasets were centered and scaled to the same standard deviation to create the final heatmap. Complete linkage hierarchical clustering with Euclidean distance was used to create the row dendrogram.

### Direct targets prediction

MAX, EP400, ST ChIP-seq data were integrated with individual differential expression data from shEP400–1, -2, -3 and shMYCL RNA-seq using Binding and Expression Target Analysis (BETA) software package, which infers activating or repressive function of MAX, EP400, ST and predict the target genes based on rank product of binding potential and differential expression [48]. Shared targets of all three factors were termed shEP400-1 BETA, shEP400-2 BETA, shEP400-3 BETA and shMYCL BETA respectively. Common targets of all four aforementioned datasets were termed BETA4, or BETA3 if shEP400-1 BETA was excluded.

## Supporting information

S1 Fig Conserved MYC boxes in MYC family proteins. **(A)** Illustration of MAX and MYC family interacting proteins highlighting interaction of ST with MYCL, MAX and EP400 complex in red boxes. (B) Conserved MYC boxes in MYCL, MYCN and MYC. (C) Predicted coding of human MYC, MYCN and MYCL isoforms i1 and i3. Conserved MYC box elements are boxed. MB0 is also known as NC1. Note that MB3a is not present in MYCL. Identical residues in red. Conserved residues in at least 2 forms in blue.
(PDF)

S2 Fig MCPyV ST mutants. A. MKL-1 cells transduced with lentiviral shRNA scrambled (shScr), LT and ST (shPanT) or ST only (shST) for 1 day followed by selection in puromycin (1 μg/ml) and cultured for 3 days were immunoblotted with Ab5 (upper panel) and Vinculin.B. Human foreskin fibroblasts (HFF) were stably transduced with lentiviruses expressing MCPyV ST, codon optimized ST (STco) or GFP. Lysates blotted with indicated antibodies.C. Alignment of MCPyV ST residues 61–109 corresponding to the region between the J domain and the Zn finger domain with ST from Gg1PyV (Gorilla gorilla gorilla 1), LIPyV (Lyon IARC, HPyV14), NJPyV (New Jersey, HPyV13), HPyV9, TSPyV (Trichodysplasia spinulosa, HPyV8), WUPyV (HPyV4), KIPyV (HPyV3), HPyV6, HPyV7, MWPyV (Malawi, HPyV10), STLPyV (Saint Louis, HPyV11), BKPyV (B.K., HPyV1), JCPyV (HPyV2) and HPyV12. The lysine residue (K61) highlighted in red is the last conserved residue in the N-terminal J domain. The cysteine residue on the right (residue 109 in MCPyV) is the first residue from the conserved Zn fingers for the ST species shown.D. HCT116 cells stably expressing MCPyV ST including wild type (WT) or indicated mutant constructs. Lysates were blotted with indicated antibodies. Input blot for ST is shown again in Fig 2D. Dashed lines are shown to distinguish lanes.
(PDF)

S3 Fig ST requires MYCL to sustain MCC viability. A. Gene Set Enrichment Analysis (GSEA) on known human housekeeping genes ranked in MKL-1 CRISPR screen using H1 (left) and H2 (right) sgRNA libraries to illustrate negative correlation of CRISPR screen and housekeeping genes.B. Copy numbers of every 50-kb segment of MKL-1 genome were called from the input of ChIP-seq experiments (see Fig 6) using QDNAseq software. Segmented copy numbers were converted to copy numbers per gene based on gene coordinates.C. Venn diagram analysis of human housekeeping genes and 481 negatively selected CRISPR targets with FDR < 0.05 identified from H1 and H2 sgRNA libraries screen of MKL-1 cells.D. Lysates from HCT116 cells stably expressing C-terminal 3xHA-tagged MYCL constructs with (+) or without (-) ST were immunoprecipitated with HA (MYCL) and Ab5 (ST) antibodies and blotted.
(PDF)

S4 Fig MAX, EP400 and MCPyV ST bind to actively transcribed promoters. A. Venn diagram of biological replicas of ChIP-seq for MAX, EP400, Ab5 and ST-HA for ST.B. Peak Height distribution. All peaks were separated into promoter, intron, and distal intragenic regions. Input Genome legend shown for comparison.C. ChIP-reChIP followed by qPCR was performed. Initial (1^st^) ChIP was performed with antibodies to MAX (left panel), EP400 (middle), ST (gray bar) and ST-HA (black) followed by re-ChIP with indicated antibody or no IgG. Primers for MCM7 or PCBP1 promoters as indicated.
(PDF)

S5 Fig Validation of ST and MAX ChIP. A. Chromatin was prepared from MKL-1 cells containing Dox inducible scrambled shRNA (shScr), MYCL (shMYCL), or Dox inducible miRNAs targeting negative control DNA sequence (mirNRneg) or MYCL (mirMYCL) after 2 days with 0.3 μg/ml Dox addition. ChIP-qPCR performed with Ab5 antibody and primers for MYCL promoter.B. Same as A with primers for indicated promoters.C. Overlapped peaks of MAX, EP400, ST and H3K4me3 ChIP-seq at MYCL locus.D. Chromatin from MKL-1 cells with a Dox inducible shRNA targeting EP400 before (Gray bars) and after (black bars) 5 days of Dox addition. ChIP-qPCR was performed with MAX antibody and indicated promoters. 544–545 and 647–648 represent two DNA sites used as negative controls.
(PDF)

S6 Fig Principal Components Analysis (PCA) plots before and after adjustment for batch effects. Principal components analysis was performed on the data before applying ComBat (but after normalization; left-hand side) and after applying ComBat (right-hand side). Colors indicate sample conditions as shown in the legend. Numbers located below each data point indicate the batch in which the experiment was performed.
(PDF)

S7 Fig MCPyV ST cooperates with MYCL and EP400 complex to activate gene expression. A. BETA Activating/Repressing Function Prediction for MAX, EP400, and ST upon EP400 or MYCL knockdown by combining MAX, EP400, ST ChIP-seq with RNA-seq from MKL-1 cells containing EP400 shRNA -1, -2, -3, shScr after 5 days Dox treatment or shMYCL after 2 days Dox treatment. Genes were Ranked on both ChIP peaks proximity to transcription start site and differential expression upon factor binding, rank product of the two was used to predict direct targets. Purple line represents genes downregulated upon EP400 knock-down (Down), red upregulated (Up) and dashed line static genes with no change. p values indicated in parentheses.B. Venn diagram showing common direct target genes of MAX, EP400 and ST identified by BETA based on ChIP-seq of MAX, EP400, ST and RNA-seq of shEP400-1, -2, -3 and MYCL shRNA.C. Venn diagram showing common direct target genes of MAX, EP400 and ST identified by BETA based on ChIP-seq of MAX, EP400, ST and RNA-seq of shEP400-1, -2, -3 and MYCL shRNA (BETA4).
(PDF)

S8 Fig Fold changes of BETA3 genes. A. Heatmap shows the logarithm (base 2) of the fold change for each BETA3 gene in each sample relative to the average expression of the same gene in the three shScr replicates in the shEP400 experiment.B. Histogram showing the spread of fold changes across all BETA3 genes in the shEP400 samples relative to the shScr samples. Fold change was computed as 2^*ΔY*^, where *ΔY* indicates the average of the log (base 2) expression levels of all six shEP400 samples (shEP400-2, -3 in triplicate) subtracted by the average log (base 2) expression levels in the three shScr (shScr in triplicate) samples from the EP400 experiment.C. Histogram showing the spread of fold changes across all BETA3 genes in the shMYCL samples relative to the shScr samples. Fold change was computed as 2^*ΔY*^, where *ΔY* indicates the average of the log (base 2) expression levels of three shMYCL samples subtracted by the average log (base 2) expression levels in the three shScr samples from the MYCL experiment.
(PDF)

S9 Fig Comparison of effect of inducible ST in IMR90 cells with depletion of EP400 and MYCL in MKL-1 cells. Heatmap illustrating comparison of all 2157 DEG genes in IMR90 cells with inducible expression of GFP or MCPyV ST with all DEG genes in MKL-1 cells after depletion of EP400 and MYCL and shScr. The IMR90 profiles were each subtracted by a corresponding control, which was defined as the average expression level in the IMR90 GFP cell line at the same time point. The MKL-1 shEP400 profiles were subtracted by the average expression level in the shScramble samples from the shEP400 batch. Likewise, the shMYCL profiles were subtracted by the average expression level in the shScramble samples from the shMYCL batch. Finally, for each gene, all its log-transformed expression values across both IMR90 and MKL-1 datasets were centered and scaled to the same standard deviation to create the final heatmap. Complete linkage hierarchical clustering with Euclidean distance was used to create the row dendrogram.
(PDF)

S1 Table MudPIT. MudPIT (Multi-dimensional protein identification technology) with antibodies to MAX, Ab5 (LT & ST), EP400, ACTL6A, Ab3 (LT only), control Ig with lysates prepared from MKL-1 and WaGa cell lines. Values indicated refer to Normalized Spectral Abundance Factor (NSAF) which represents the fraction of total immunoprecipitate that was represented by the number of peptides assigned to a given protein. Targets are grouped are shown in Fig 1B.
(PDF)

S2 Table CRISPR. Tabs
MKL-1 Copy number variationCRISPR targets FDR 0.05EP400 componentsHousekeeping
(XLSX)

S3 Table Expression Profiling. Counts were normalized separately for the two experiments (shEP400 and shMYCL) using “voom” from the R/Bioconductor package *limma* (VoomSeparate) then corrected for batch effect using ComBat (CB). DEG refers to genes that were differentially expressed with *P*_adj_ < 0.001 in all comparisons (shEP400-2, shEP400-3 and shMYCL relative to shScr). BETA3 represents 951 genes downregulated by shEP400 and shMYCL and identified by ChIP-seq of ST, MAX and EP400. Genes were clustered using model-based clustering (mclust) and merged into larger patterns using hierarchical clustering (hmcl). Gene Ontology (GO) term enrichment is presented in files labeled GO. Files #3 and #6 provide the full mapping between cluster numbers and genes for DEG and BETA3, respectively.
VoomSeparateCB_DEG_hmcl_GOVoomSeparateCB_DEG_mclust_GOVoomSeparateCB_DEG_mclust_mergeVoomSeparateCB_BETA3_hmcl_GOVoomSeparateCB_BETA3_mclust_GOVoomSeparateCB_BETA3_mclust_mer
(XLSX)

S4 Table BETA. MAX ChIP_shEP400-1EP400 ChIP_shEP400-1ST ChIP_shEP400-1Shared Targets_shEP400-1 BETAMAX ChIP_shEP400-2EP400 ChIP_shEP400-2ST ChIP_shEP400-2Shared Targets_shEP400-2 BETAMAX ChIP_shEP400-3EP400 ChIP_shEP400-3ST ChIP_shEP400-3Shared Targets_shEP400-3 BETAMAX ChIP_shMYCLEP400 ChIP_shMYCLST ChIP_shMYCLShared Targets_shMYCL BETABETA4BETA3
(XLSX)

S5 Table Fold changes of the BETA3 genes in response to shEP400 and shMYCL. (XLSX)
