# Supplementary figures and images for: Merkel cell polyomavirus recruits MYCL to the EP400 complex to promote oncogenesis
Source: PLoS Pathog. 2017 Oct 13;13(10):e1006668. doi: 10.1371/journal.ppat.1006668 (PMC5640240; doi:10.1371/journal.ppat.1006668)

S1 Fig. Conserved MYC boxes in MYC family proteins

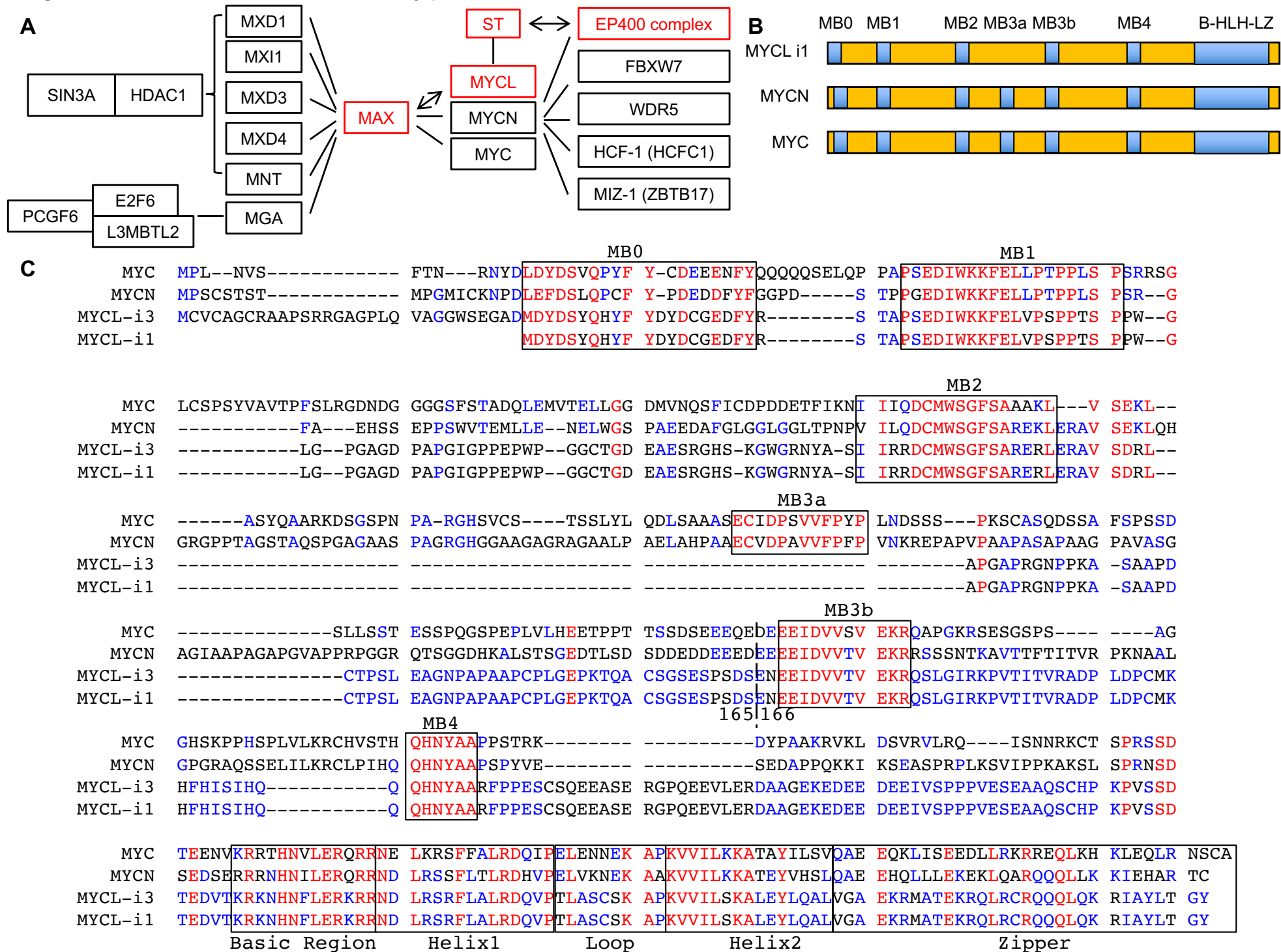

Supplement: S1 Fig — (A) Illustration of MAX and MYC family interacting proteins highlighting interaction of ST with MYCL, MAX and EP400 complex in red boxes. (B) Conserved MYC boxes in MYCL, MYCN and MYC. (C) Predicted coding of human MYC, MYCN and MYCL isoforms i1 and i3. Conserved MYC box elements are boxed. MB0 is also known as NC1. Note that MB3a is not present in MYCL. Identical residues in red. Conserved residues in at least 2 forms in blue. (PDF) [file ppat.1006668.s001.pdf]

S3 Fig. ST requires MYCL to sustain MCC viability

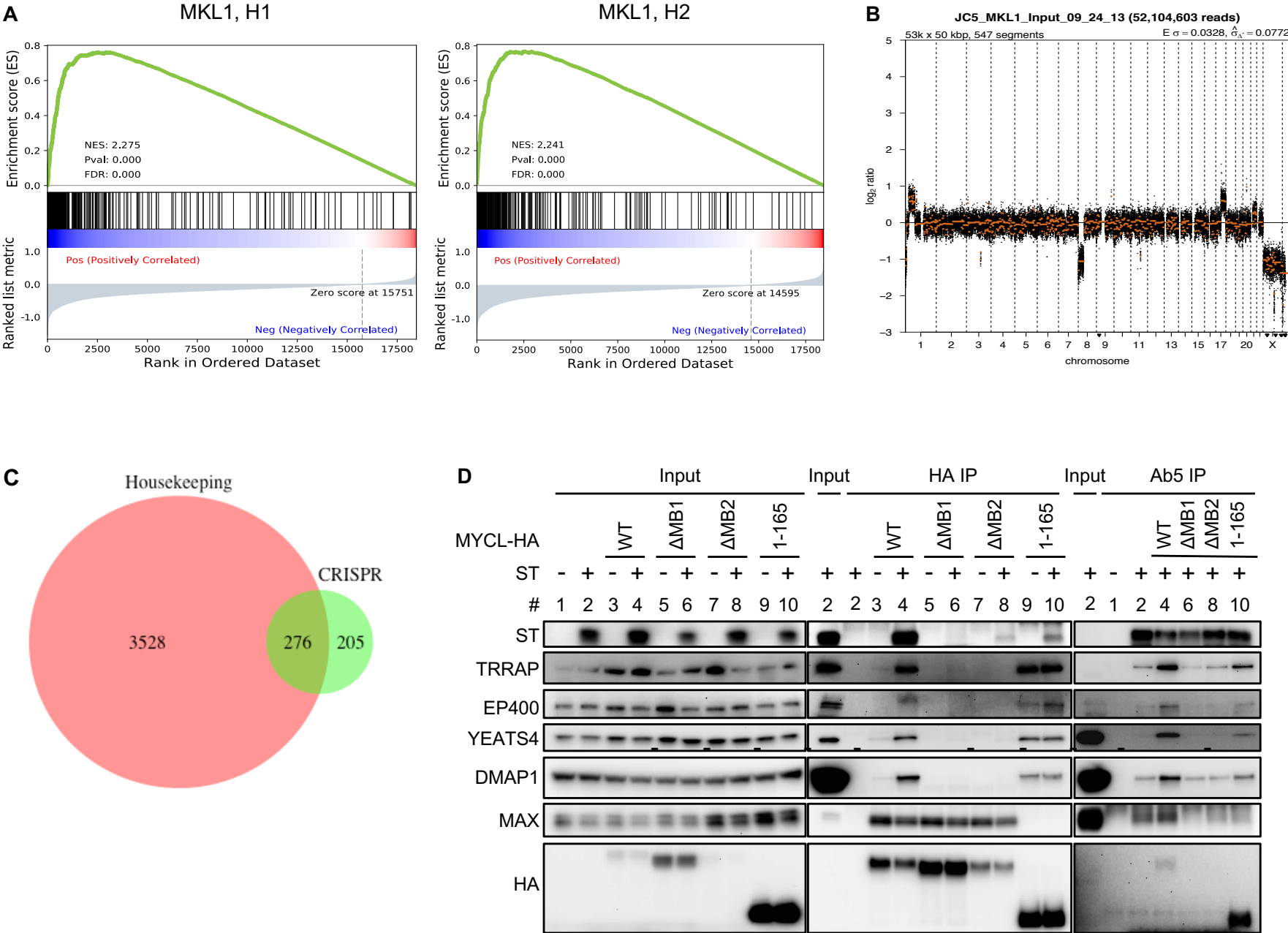

Supplement: S3 Fig — A. Gene Set Enrichment Analysis (GSEA) on known human housekeeping genes ranked in MKL-1 CRISPR screen using H1 (left) and H2 (right) sgRNA libraries to illustrate negative correlation of CRISPR screen and housekeeping genes.B. Copy numbers of every 50-kb segment of MKL-1 genome were called from the input of ChIP-seq experiments (see Fig 6) using QDNAseq software. Segmented copy numbers were converted to copy numbers per gene based on gene coordinates.C. Venn diagram analysis of human housekeeping genes and 481 negatively selected CRISPR targets with FDR < 0.05 identified from H1 and H2 sgRNA libraries screen of MKL-1 cells.D. Lysates from HCT116 cells stably expressing C-terminal 3xHA-tagged MYCL constructs with (+) or without (-) ST were immunoprecipitated with HA (MYCL) and Ab5 (ST) antibodies and blotted. (PDF) [file ppat.1006668.s003.pdf]

S4 Fig. MAX, EP400 and MCPyV ST bind to actively transcribed promoters

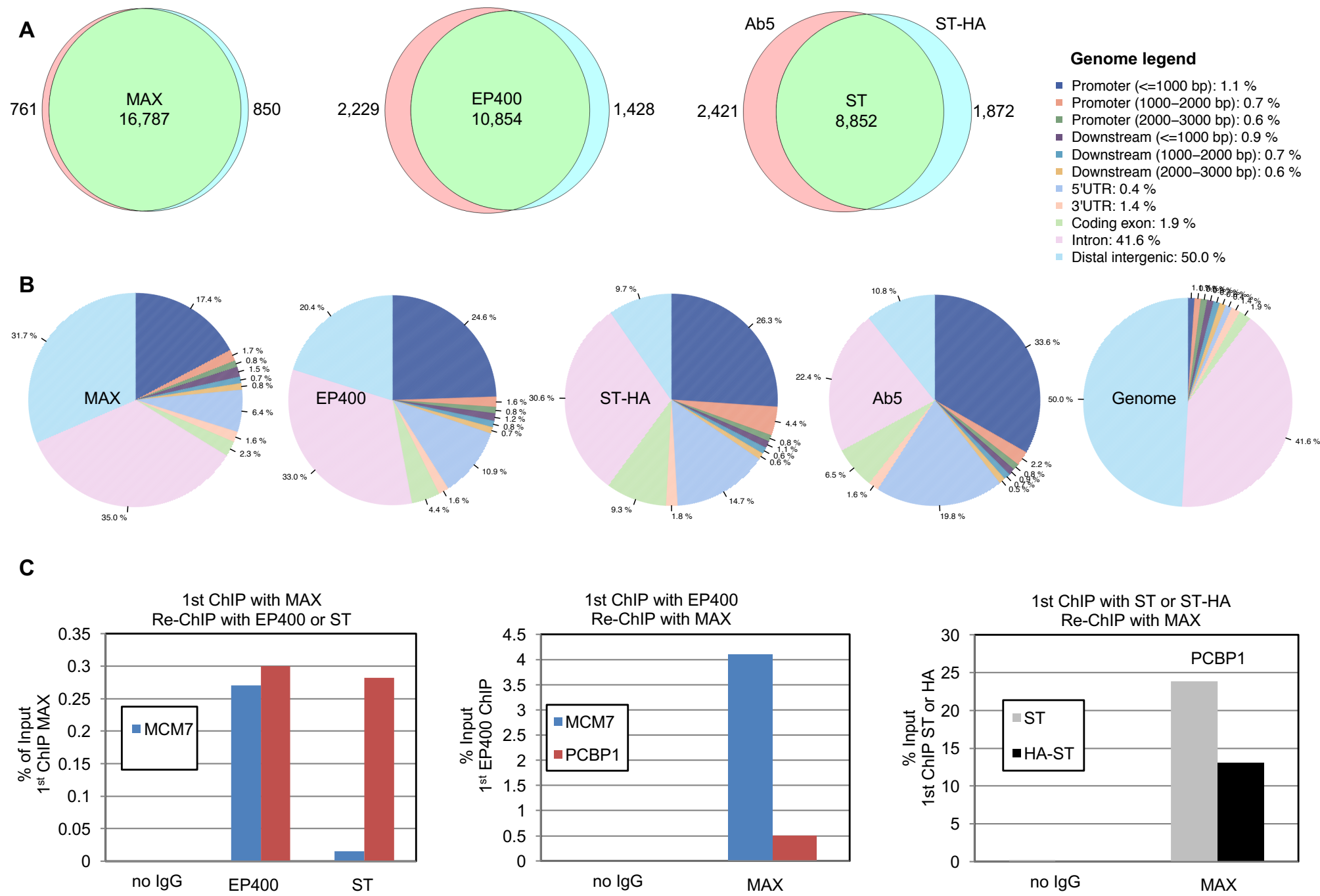

Supplement: S4 Fig — A. Venn diagram of biological replicas of ChIP-seq for MAX, EP400, Ab5 and ST-HA for ST.B. Peak Height distribution. All peaks were separated into promoter, intron, and distal intragenic regions. Input Genome legend shown for comparison.C. ChIP-reChIP followed by qPCR was performed. Initial (1st) ChIP was performed with antibodies to MAX (left panel), EP400 (middle), ST (gray bar) and ST-HA (black) followed by re-ChIP with indicated antibody or no IgG. Primers for MCM7 or PCBP1 promoters as indicated. (PDF) [file ppat.1006668.s004.pdf]

**S5 Fig. Validation of ST and MAX ChIP.**

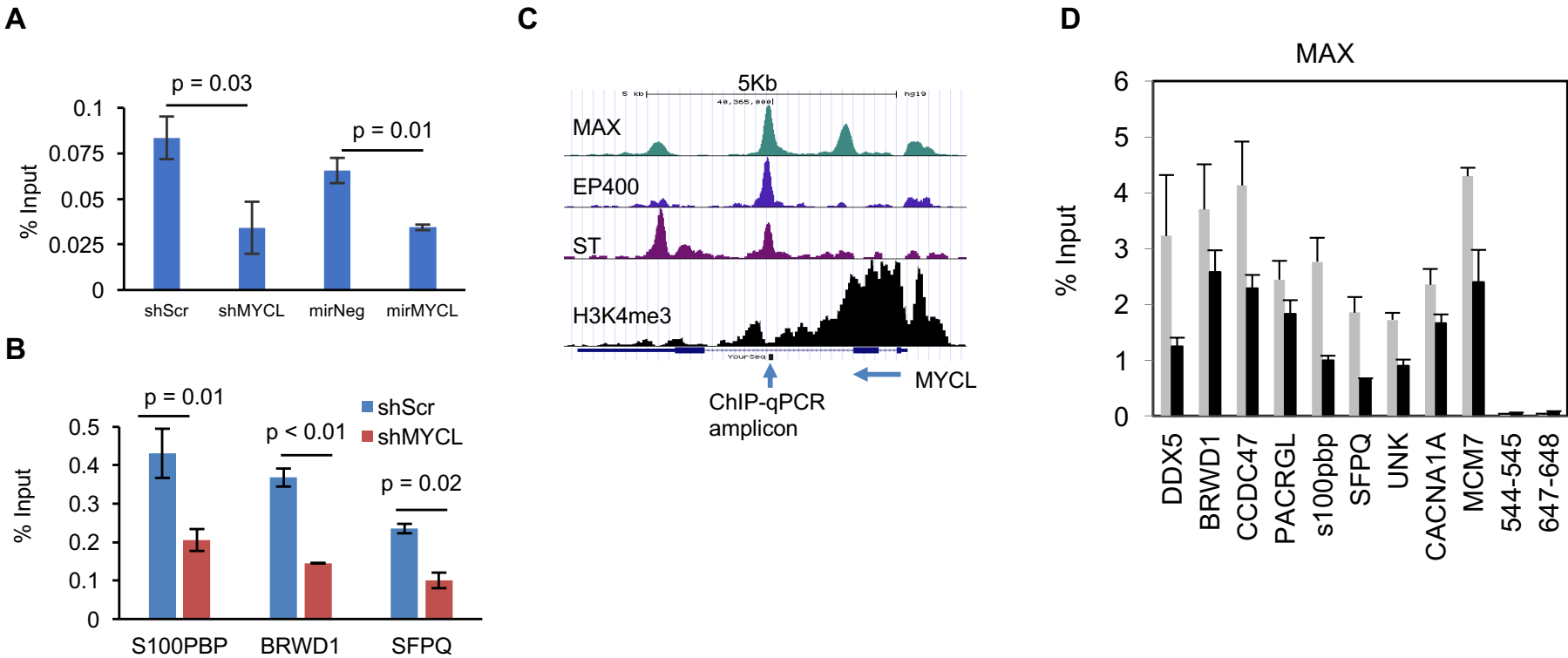

Supplement: S5 Fig — A. Chromatin was prepared from MKL-1 cells containing Dox inducible scrambled shRNA (shScr), MYCL (shMYCL), or Dox inducible miRNAs targeting negative control DNA sequence (mirNRneg) or MYCL (mirMYCL) after 2 days with 0.3 μg/ml Dox addition. ChIP-qPCR performed with Ab5 antibody and primers for MYCL promoter.B. Same as A with primers for indicated promoters.C. Overlapped peaks of MAX, EP400, ST and H3K4me3 ChIP-seq at MYCL locus.D. Chromatin from MKL-1 cells with a Dox inducible shRNA targeting EP400 before (Gray bars) and after (black bars) 5 days of Dox addition. ChIP-qPCR was performed with MAX antibody and indicated promoters. 544–545 and 647–648 represent two DNA sites used as negative controls. (PDF) [file ppat.1006668.s005.pdf]

S6 Fig. Principal Components Analysis (PCA) plots before and after adjustment for batch effects

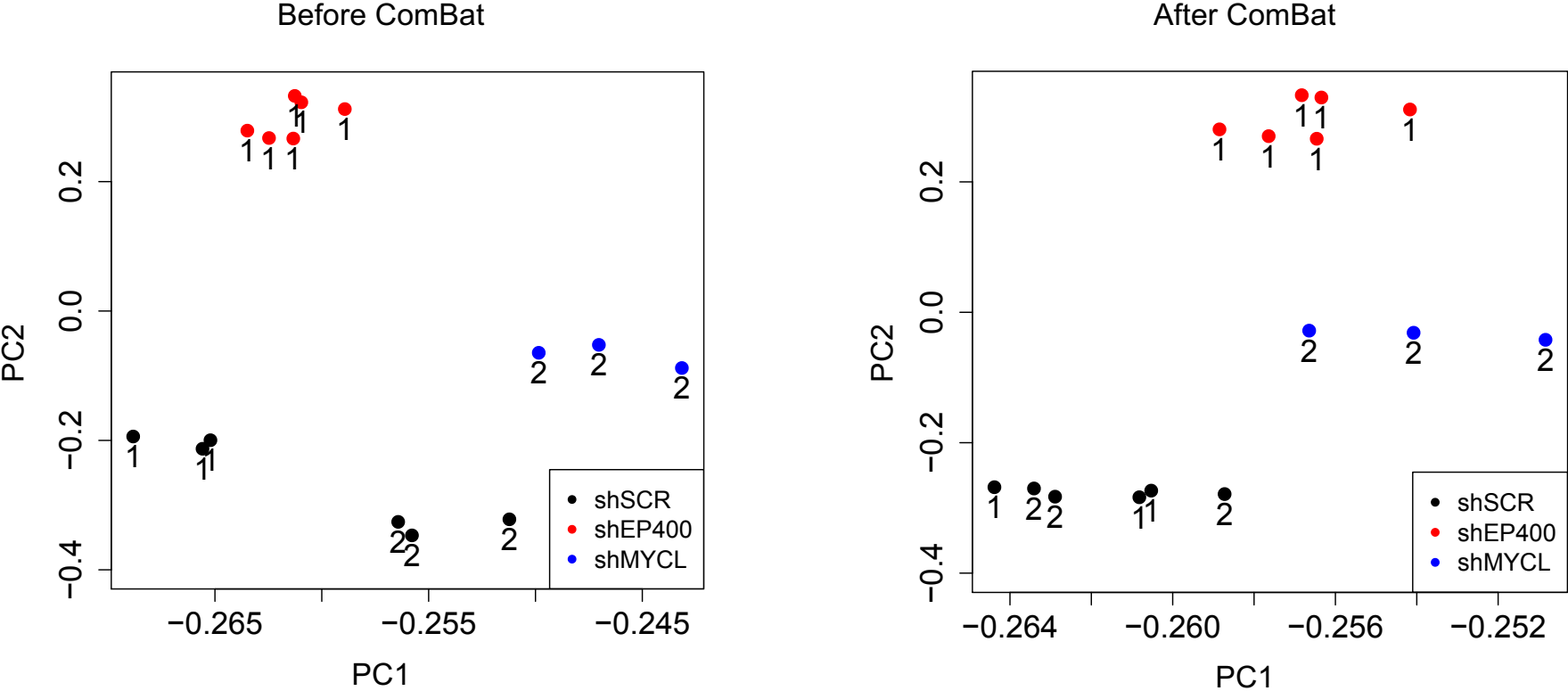

Supplement: S6 Fig — Principal components analysis was performed on the data before applying ComBat (but after normalization; left-hand side) and after applying ComBat (right-hand side). Colors indicate sample conditions as shown in the legend. Numbers located below each data point indicate the batch in which the experiment was performed. (PDF) [file ppat.1006668.s006.pdf]

S8 Fig. Fold changes of BETA3 genes.

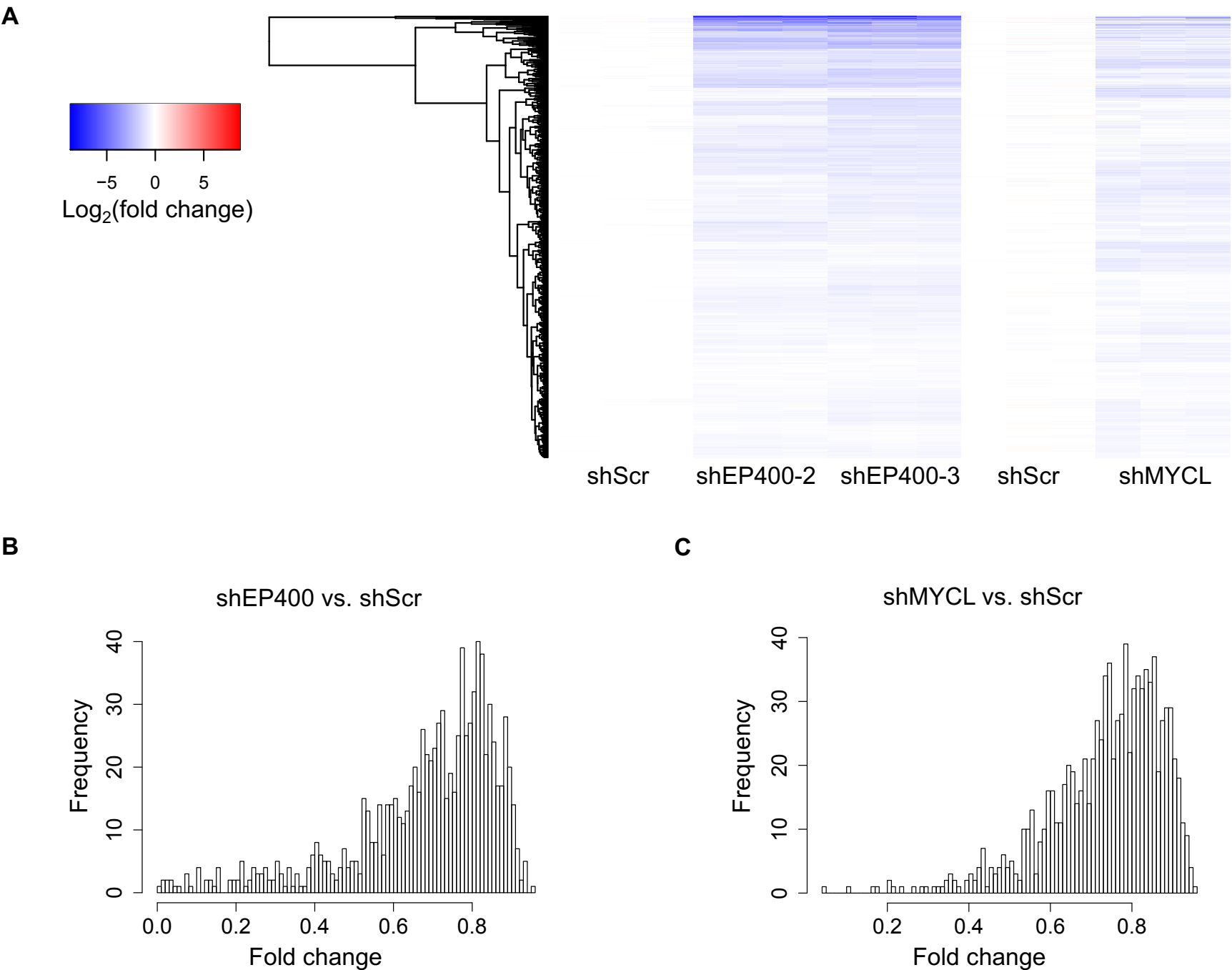

Supplement: S8 Fig — A. Heatmap shows the logarithm (base 2) of the fold change for each BETA3 gene in each sample relative to the average expression of the same gene in the three shScr replicates in the shEP400 experiment.B. Histogram showing the spread of fold changes across all BETA3 genes in the shEP400 samples relative to the shScr samples. Fold change was computed as 2ΔY, where ΔY indicates the average of the log (base 2) expression levels of all six shEP400 samples (shEP400-2, -3 in triplicate) subtracted by the average log (base 2) expression levels in the three shScr (shScr in triplicate) samples from the EP400 experiment.C. Histogram showing the spread of fold changes across all BETA3 genes in the shMYCL samples relative to the shScr samples. Fold change was computed as 2ΔY, where ΔY indicates the average of the log (base 2) expression levels of three shMYCL samples subtracted by the average log (base 2) expression levels in the three shScr samples from the MYCL experiment. (PDF) [file ppat.1006668.s008.pdf]

**S9 Fig. Comparison of effect of inducible ST in IMR90 cells with depletion of EP400 and MYCL in MKL-1 cells**

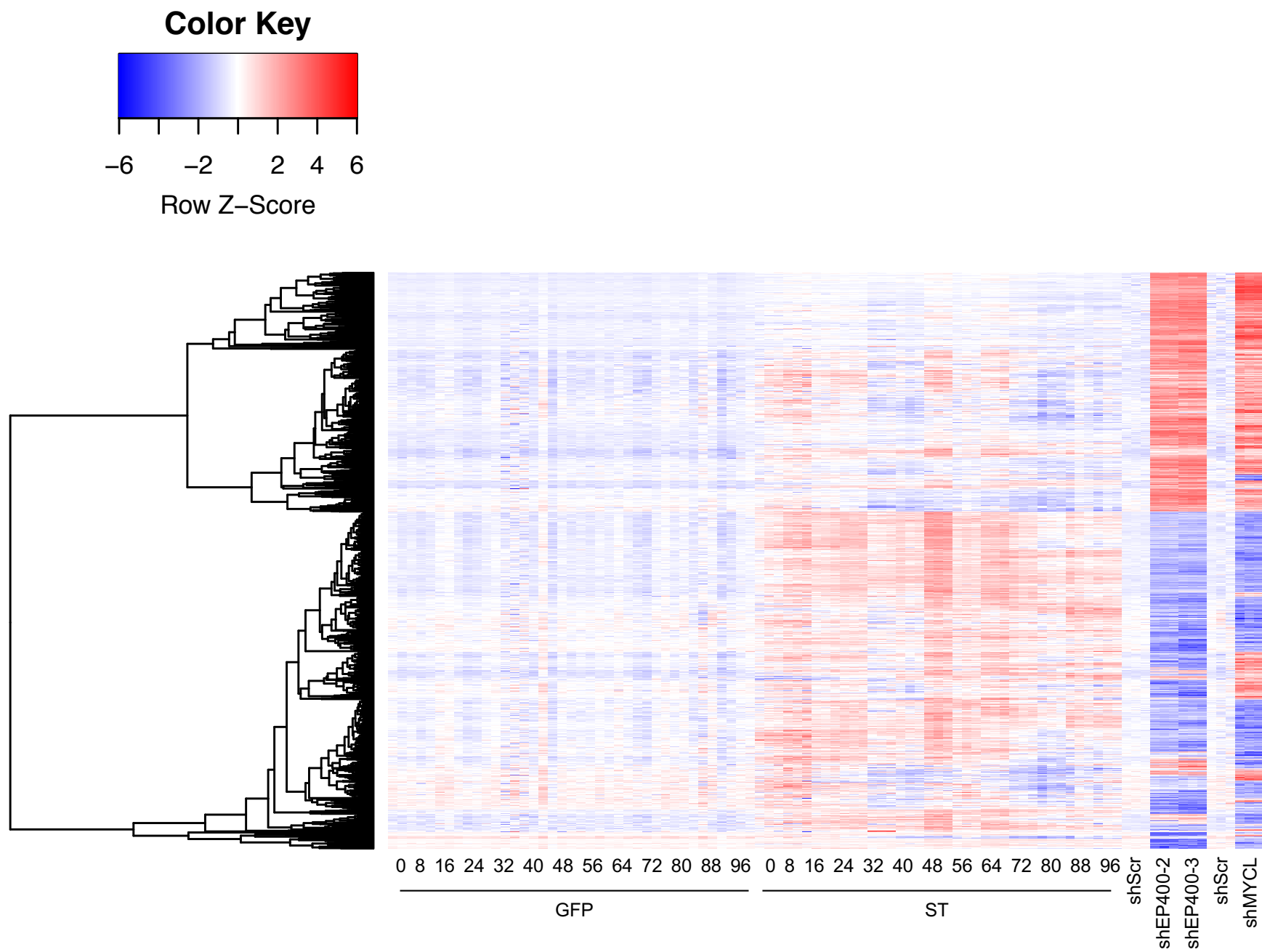

Supplement: S9 Fig — Heatmap illustrating comparison of all 2157 DEG genes in IMR90 cells with inducible expression of GFP or MCPyV ST with all DEG genes in MKL-1 cells after depletion of EP400 and MYCL and shScr. The IMR90 profiles were each subtracted by a corresponding control, which was defined as the average expression level in the IMR90 GFP cell line at the same time point. The MKL-1 shEP400 profiles were subtracted by the average expression level in the shScramble samples from the shEP400 batch. Likewise, the shMYCL profiles were subtracted by the average expression level in the shScramble samples from the shMYCL batch. Finally, for each gene, all its log-transformed expression values across both IMR90 and MKL-1 datasets were centered and scaled to the same standard deviation to create the final heatmap. Complete linkage hierarchical clustering with Euclidean distance was used to create the row dendrogram. (PDF) [file ppat.1006668.s009.pdf]
