# Supplementary material for: Merkel cell polyomavirus recruits MYCL to the EP400 complex to promote oncogenesis
Source: PLoS Pathog. 2017 Oct 13;13(10):e1006668. doi: 10.1371/journal.ppat.1006668 (PMC5640240; doi:10.1371/journal.ppat.1006668)
Supplement: S2 Fig — A. MKL-1 cells transduced with lentiviral shRNA scrambled (shScr), LT and ST (shPanT) or ST only (shST) for 1 day followed by selection in puromycin (1 μg/ml) and cultured for 3 days were immunoblotted with Ab5 (upper panel) and Vinculin.B. Human foreskin fibroblasts (HFF) were stably transduced with lentiviruses expressing MCPyV ST, codon optimized ST (STco) or GFP. Lysates blotted with indicated antibodies.C. Alignment of MCPyV ST residues 61–109 corresponding to the region between the J domain and the Zn finger domain with ST from Gg1PyV (Gorilla gorilla gorilla 1), LIPyV (Lyon IARC, HPyV14), NJPyV (New Jersey, HPyV13), HPyV9, TSPyV (Trichodysplasia spinulosa, HPyV8), WUPyV (HPyV4), KIPyV (HPyV3), HPyV6, HPyV7, MWPyV (Malawi, HPyV10), STLPyV (Saint Louis, HPyV11), BKPyV (B.K., HPyV1), JCPyV (HPyV2) and HPyV12. The lysine residue (K61) highlighted in red is the last conserved residue in the N-terminal J domain. The cysteine residue on the right (residue 109 in MCPyV) is the first residue from the conserved Zn fingers for the ST species shown.D. HCT116 cells stably expressing MCPyV ST including wild type (WT) or indicated mutant constructs. Lysates were blotted with indicated antibodies. Input blot for ST is shown again in Fig 2D. Dashed lines are shown to distinguish lanes. (PDF) [file ppat.1006668.s002.pdf]

S2 Fig. MCPyV ST mutants

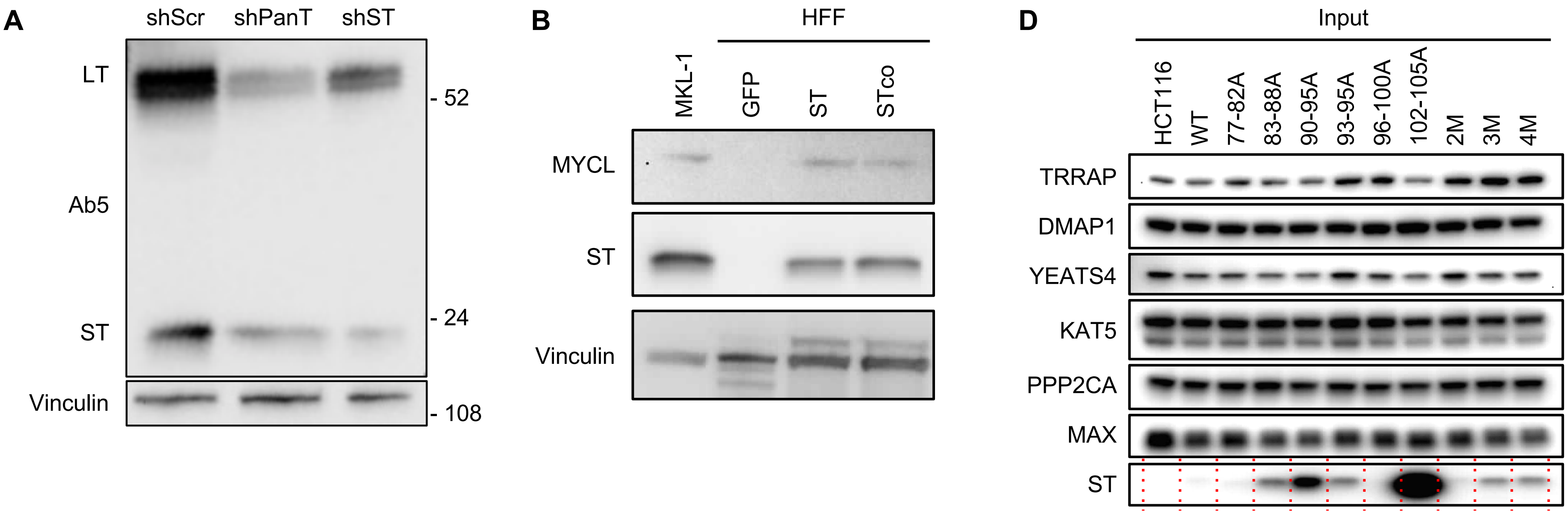

C

```
          61                                                86     87             92,93                               109
  MCPyV  KFQ--Q-NIH KL-R-S-DF- S-MF--D--E V-STKF-PWE ---E-----Y -GTL-KD-YM QSG----Y-N AR-FCRG--S -K-C
Gorilla  KFQ--R-NIH KL-R-S-EF- T-IF--D--E V-SIKF-PWE ---E-----Y -GTL-KD-YM QSG----Y-R AI-FCKG--P -G-C
  LIPyV  KFT--T-SLT EA-R-A-S-- T--Y-----Q A-STLF--WE I--D------ -NPL-KN-LL --GP---VIK -RPFLKS--P -H-C
  NJPyV  KFQ--E-GLI EV-RDS-E-- V--C-----Q V-S--F---S ---DC----Y DSSLLK--CC -S-PK--VFH EL-FLRS--P -Q-C
  HPyV9  KLQ--V-TLL EI-R-S-NCG S---S-SS-Q V-A-WYF-WD E--N-----F -RTL-GA-FL --GEK--F-N QR-IIGG-YP -D-C
  TSPyV  KLQ--E-GIY NA-R-Q-EFP T---SFSS-Q VGS-WY--WE A--N-----L -ISL-KE-YF --GKKK-Y-D EN-VIKH-WP -Q-C
  WUPyV  KLQECVSTVH QL-N-E-EED E-VWS-SS-Q V----EC-TE LCCNFPPRKY -R-LVGEVY- --GDV--F-E EY-ILKD-W- -DIC
  KIPyV  KLQDSVSSVH DL-N-E-EED N-IWQ-SS-Q V----YC-KD LCCN----KF -R-LVGAIY- --GDY--Y-E AY-IMKQ-W- -DVC
  HPyV6  KLN--A-T-- -L-RDQ-MSS SPTWCFSS-E V-S--D-DWG I--P-----L --TV-GE-FL --GPE--FHK RK-V----WD FRLC
  HPyV7  KLE--A-T-- -L-RDQ-RSG SPMWHYSSDE V-S--F--WD I--E-----L --TV-GE-FL --GPE--FNR KK-V----WN YNLC
  MWPyV  KMY--T-TIE KL-R---REG E-VY-FPA-K V-G-YFID-D V--------V --TL-GD-VL --GPS--F-E EK-II-YIWP --LC
 STLPyV  RMQ--N-TLQ NL-RSS-NEN ENMY-PPV-R M---LLLT-D T--------F --TL-GE-LL --GPQ--F-E SK-VI-FIWP --TC
  BKPyV  KME--Q-DV- KVAH-QPDFG T--WS-SS-E V-----CA-D ---------F --PL-----C ---P-----D TL-YCKE-WP -I-C
  JCPyV  KME--Q-GV- KVAH-QPDFG T--WN-SS-E V-G---C--D ---------F --P------- ---PN--S-D TL-YCKE-WP -N-C
 HPyV12  KFK--D-GIY NL-R-EVK-P S-LH--PV-- V-T---C--- ---------- --TV-----L --GARNIF-N -L-ITNS--S -Q-C
```
