# Supplementary material for: Merkel cell polyomavirus recruits MYCL to the EP400 complex to promote oncogenesis
Source: PLoS Pathog. 2017 Oct 13;13(10):e1006668. doi: 10.1371/journal.ppat.1006668 (PMC5640240; doi:10.1371/journal.ppat.1006668)

S7 Fig. MCPyV ST cooperates with MYCL and EP400 complex to activate gene expression

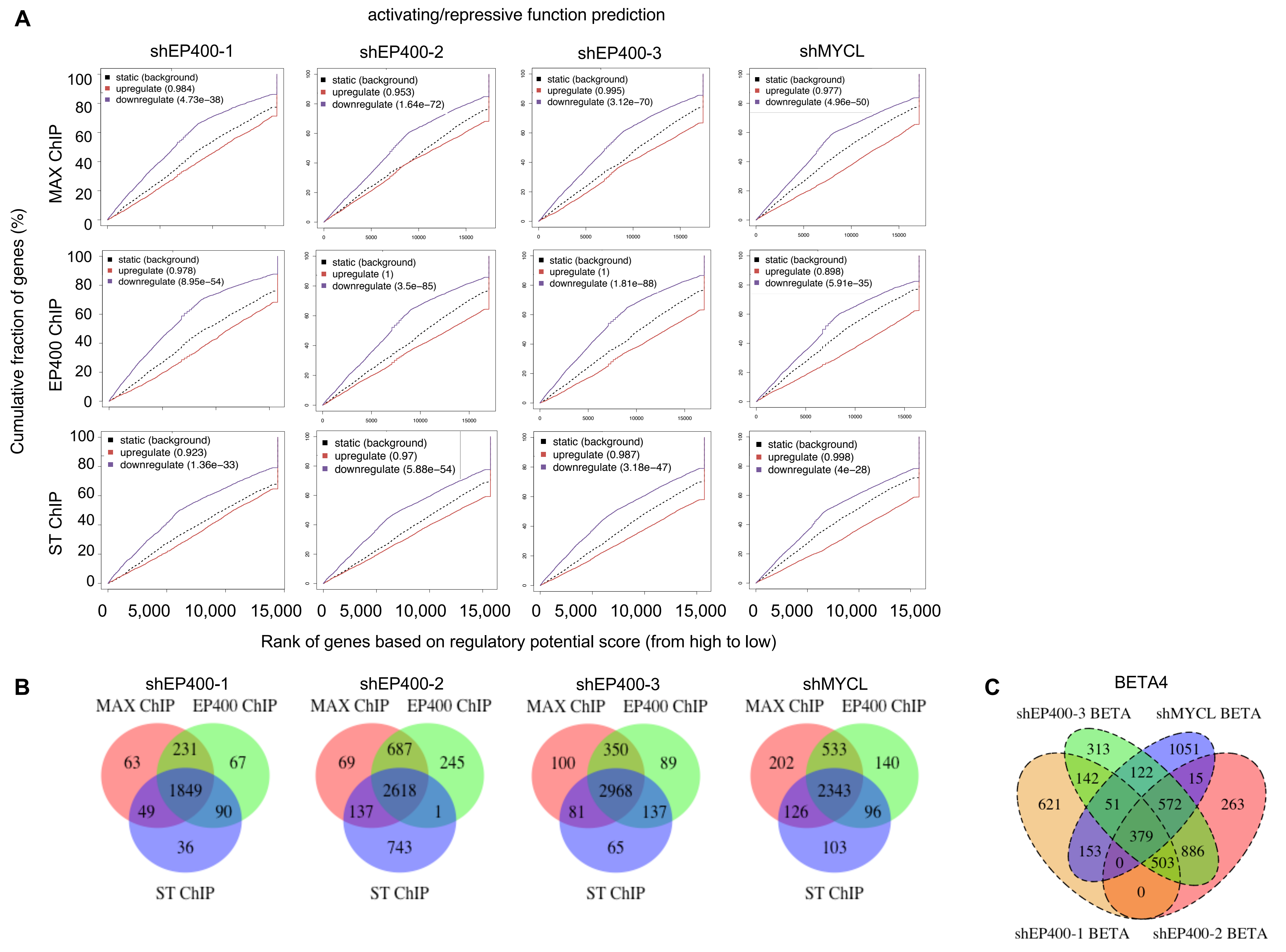

Supplement: S7 Fig — A. BETA Activating/Repressing Function Prediction for MAX, EP400, and ST upon EP400 or MYCL knockdown by combining MAX, EP400, ST ChIP-seq with RNA-seq from MKL-1 cells containing EP400 shRNA -1, -2, -3, shScr after 5 days Dox treatment or shMYCL after 2 days Dox treatment. Genes were Ranked on both ChIP peaks proximity to transcription start site and differential expression upon factor binding, rank product of the two was used to predict direct targets. Purple line represents genes downregulated upon EP400 knock-down (Down), red upregulated (Up) and dashed line static genes with no change. p values indicated in parentheses.B. Venn diagram showing common direct target genes of MAX, EP400 and ST identified by BETA based on ChIP-seq of MAX, EP400, ST and RNA-seq of shEP400-1, -2, -3 and MYCL shRNA.C. Venn diagram showing common direct target genes of MAX, EP400 and ST identified by BETA based on ChIP-seq of MAX, EP400, ST and RNA-seq of shEP400-1, -2, -3 and MYCL shRNA (BETA4). (PDF) [file ppat.1006668.s007.pdf]
