# Supplementary material for: Merkel cell polyomavirus recruits MYCL to the EP400 complex to promote oncogenesis
Source: PLoS Pathog. 2017 Oct 13;13(10):e1006668. doi: 10.1371/journal.ppat.1006668 (PMC5640240; doi:10.1371/journal.ppat.1006668)
Supplement: S1 Table — MudPIT (Multi-dimensional protein identification technology) with antibodies to MAX, Ab5 (LT & ST), EP400, ACTL6A, Ab3 (LT only), control Ig with lysates prepared from MKL-1 and WaGa cell lines. Values indicated refer to Normalized Spectral Abundance Factor (NSAF) which represents the fraction of total immunoprecipitate that was represented by the number of peptides assigned to a given protein. Targets are grouped are shown in Fig 1B. (PDF) [file ppat.1006668.s010.pdf]

Supplementary Table 1. MudPIT

		MAX					Ab5				EP400			Ab3		IgG			
**Group**	**Gene**	**MKL-1**	**MKL-1**	**MKL-1**	**MKL-1**	**MKL-1**	**MKL-1**	**MKL-1**	**MKL-1**	**WaGa**	**WaGa**	**MKL-1**	**MKL-1**	**MKL-1**	**WaGa**	**MKL-1**	**MKL-1**	**WaGa**	**All**
**LT**	MCV LT	0.00008	0.00000	0.00000	0.00000	0.00000	0.06235	0.03120	0.01272	0.06183	0.00000	0.00012	0.00024	0.03213	0.01710	0.00020	0.00012	0.00000	0.00589
	VPS39	0.00000	0.00000	0.00000	0.00000	0.00000	0.00756	0.00013	0.00033	0.00560	0.00000	0.00000	0.00000	0.00020	0.00143	0.00000	0.00000	0.00000	0.00021
	RB1	0.00000	0.00000	0.00000	0.00000	0.00000	0.00000	0.00000	0.00000	0.00141	0.00000	0.00000	0.00000	0.00004	0.00000	0.00000	0.00000	0.00000	0.00001
**ST**	MCV ST	0.00094	0.00386	0.00150	0.00201	0.00257	0.00058	0.00092	0.00084	0.01989	0.00000	0.00045	0.00000	0.00000	0.00000	0.00000	0.00000	0.00000	0.00053
	PPP2R1A	0.00800	0.00382	0.01939	0.00812	0.01242	0.02074	0.00104	0.00084	0.02109	0.00051	0.00187	0.00119	0.00000	0.00051	0.00000	0.00000	0.00000	0.00564
	PPP2CA	0.00475	0.00111	0.00037	0.01301	0.00372	0.01433	0.00000	0.00084	0.00529	0.00194	0.00083	0.00040	0.00000	0.00010	0.00000	0.00000	0.00000	0.00312
	PPP2CB	0.00045	0.00111	0.00037	0.00000	0.00053	0.01433	0.00000	0.00000	0.00529	0.00194	0.00000	0.00000	0.00000	0.00010	0.00000	0.00000	0.00000	0.00030
	PPP2R1B	0.00055	0.00013	0.00061	0.00051	0.00069	0.00102	0.00001	0.00000	0.00387	0.00000	0.00000	0.00000	0.00000	0.00000	0.00000	0.00000	0.00000	0.00030
**MAX**	MAX	0.04584	0.00477	0.02878	0.03100	0.01449	0.00827	0.00000	0.00000	0.00409	0.00000	0.00032	0.00000	0.00000	0.00000	0.00000	0.00000	0.00000	0.00642
	MYCL	0.00203	0.00119	0.00341	0.00466	0.00113	0.00207	0.00000	0.00000	0.00913	0.00000	0.00019	0.00031	0.00000	0.00000	0.00000	0.00000	0.00000	0.00135
	MYCN	0.00008	0.00000	0.00000	0.00000	0.00000	0.00110	0.00000	0.00000	0.00000	0.00000	0.00000	0.00000	0.00000	0.00000	0.00000	0.00000	0.00000	0.00004
**EP400**	RUVBL2	0.00436	0.00055	0.00537	0.00264	0.01828	0.02617	0.00114	0.00134	0.00989	0.00194	0.00199	0.00018	0.00008	0.00052	0.00037	0.00022	0.00000	0.00348
	TRRAP	0.00516	0.00360	0.00306	0.00468	0.00297	0.02124	0.00002	0.00001	0.01101	0.00264	0.00048	0.00039	0.00000	0.00000	0.00000	0.00000	0.00000	0.00275
	ACTL6A	0.01219	0.01419	0.00278	0.01113	0.00638	0.01718	0.00374	0.00006	0.00490	0.00280	0.00164	0.00076	0.00000	0.00028	0.00000	0.00000	0.00000	0.00605
	ACTL6B	0.00778	0.00139	0.00191	0.00084	0.00289	0.00755	0.00049	0.00000	0.00197	0.00141	0.00021	0.00030	0.00000	0.00028	0.00000	0.00012	0.00000	0.00323
	EP400	0.00279	0.00137	0.00132	0.00248	0.00148	0.01034	0.00003	0.00001	0.00419	0.00596	0.00087	0.00034	0.00000	0.00000	0.00000	0.00000	0.00000	0.00147
	MEAF6	0.00845	0.00205	0.00204	0.01304	0.00750	0.03495	0.00000	0.00000	0.00651	0.01045	0.00013	0.00082	0.00000	0.00000	0.00000	0.00025	0.00000	0.00451
	MRGBP	0.00389	0.00461	0.00760	0.00225	0.00137	0.01098	0.00000	0.00000	0.00641	0.00000	0.00075	0.00020	0.00000	0.00000	0.00000	0.00000	0.00000	0.00241
	KAT5	0.00355	0.00036	0.00074	0.00111	0.00226	0.01072	0.00000	0.00000	0.00596	0.00364	0.00057	0.00008	0.00000	0.00000	0.00000	0.00000	0.00000	0.00143
	YEATS4	0.00223	0.00147	0.00151	0.00202	0.00295	0.00942	0.00000	0.00000	0.00144	0.00000	0.00045	0.00036	0.00000	0.00000	0.00000	0.00000	0.00000	0.00126
	MORF4L1	0.00169	0.01031	0.00028	0.00041	0.00219	0.00694	0.00000	0.00000	0.00051	0.00000	0.00016	0.00006	0.00000	0.00000	0.00000	0.00000	0.00000	0.00155
	DMAP1	0.00156	0.00302	0.00215	0.00196	0.00114	0.00501	0.00000	0.00000	0.00770	0.00128	0.00088	0.00035	0.00000	0.00000	0.00000	0.00000	0.00000	0.00119
	BRD8	0.00634	0.00080	0.01534	0.00420	0.00235	0.00364	0.00000	0.00000	0.00481	0.00032	0.00057	0.00026	0.00000	0.00000	0.00000	0.00000	0.00000	0.00329
	EPC2	0.00145	0.00005	0.00059	0.00016	0.00083	0.00240	0.00000	0.00000	0.00284	0.00260	0.00041	0.00036	0.00000	0.00000	0.00000	0.00000	0.00000	0.00057
	MORF4L2	0.00088	0.00054	0.00000	0.00046	0.00085	0.00177	0.00000	0.00000	0.00000	0.00000	0.00000	0.00029	0.00000	0.00000	0.00000	0.00000	0.00000	0.00044
	EPC1	0.00073	0.00052	0.00101	0.00039	0.00037	0.00158	0.00000	0.00000	0.00000	0.00144	0.00018	0.00015	0.00000	0.00000	0.00000	0.00000	0.00000	0.00039
	MBTD1	0.00064	0.00028	0.00138	0.00191	0.00036	0.00162	0.00000	0.00000	0.00729	0.00000	0.00049	0.00026	0.00000	0.00000	0.00000	0.00000	0.00000	0.00054
	ING3	0.00185	0.00155	0.00136	0.00355	0.00120	0.00049	0.00000	0.00000	0.00782	0.00144	0.00080	0.00010	0.00000	0.00000	0.00000	0.00000	0.00000	0.00113
	VPS72	0.00007	0.00000	0.00044	0.00000	0.00061	0.00364	0.00000	0.00000	0.00000	0.00000	0.00021	0.00045	0.00000	0.00000	0.00000	0.00000	0.00000	0.00017
	RUVBL1	0.00431	0.00094	0.00105	0.00690	0.02113	0.01451	0.00128	0.00119	0.04302	0.00263	0.00219	0.00036	0.00038	0.00000	0.00075	0.00000	0.00110	0.00394
**MAD**	E2F6	0.00408	0.00000	0.00154	0.00676	0.00268	0.00000	0.00000	0.00000	0.00000	0.00000	0.00000	0.00000	0.00000	0.00000	0.00000	0.00000	0.00000	0.00174
	TFDP1	0.00266	0.00043	0.00339	0.00229	0.00129	0.00174	0.00000	0.00000	0.00000	0.00000	0.00000	0.00000	0.00017	0.00044	0.00000	0.00000	0.00000	0.00120
	MGA	0.00113	0.00171	0.00257	0.00061	0.00077	0.00000	0.00000	0.00000	0.00000	0.00000	0.00000	0.00000	0.00002	0.00002	0.00000	0.00000	0.00000	0.00075
	PCGF6	0.00578	0.00923	0.00201	0.00094	0.00239	0.00000	0.00000	0.00000	0.00000	0.00000	0.00000	0.00000	0.00010	0.00017	0.00000	0.00000	0.00000	0.00270
	RING1	0.00244	0.00092	0.00067	0.00086	0.00088	0.00000	0.00000	0.00000	0.00000	0.00000	0.00000	0.00000	0.00000	0.00000	0.00000	0.00000	0.00000	0.00084
	CBX3	0.00272	0.00000	0.00160	0.00298	0.00076	0.00000	0.00000	0.00000	0.00000	0.00000	0.00000	0.00000	0.00019	0.00000	0.00000	0.00000	0.00000	0.00104
	RNF2	0.00158	0.00012	0.00122	0.00124	0.00043	0.00000	0.00000	0.00000	0.00000	0.00000	0.00000	0.00000	0.00000	0.00000	0.00000	0.00000	0.00000	0.00061
	L3MBTL2	0.00039	0.00097	0.00116	0.00028	0.00028	0.00000	0.00000	0.00000	0.00000	0.00000	0.00000	0.00000	0.00005	0.00000	0.00000	0.00000	0.00000	0.00029
	RYBP	0.00110	0.00000	0.00050	0.00000	0.00000	0.00000	0.00000	0.00000	0.00000	0.00000	0.00000	0.00000	0.00000	0.00000	0.00000	0.00000	0.00000	0.00031
	YAF2	0.00011	0.00000	0.00117	0.00000	0.00000	0.00000	0.00000	0.00000	0.00000	0.00000	0.00000	0.00000	0.00000	0.00000	0.00000	0.00000	0.00000	0.00011
	MXD1	0.00064	0.00000	0.00093	0.00010	0.00000	0.00000	0.00000	0.00000	0.00000	0.00000	0.00000	0.00000	0.00000	0.00000	0.00000	0.00000	0.00000	0.00023
	MXD3	0.00045	0.00010	0.00111	0.00000	0.00000	0.00000	0.00000	0.00000	0.00000	0.00000	0.00000	0.00000	0.00000	0.00000	0.00000	0.00000	0.00000	0.00032
	MXD4	0.00046	0.00009	0.00087	0.00000	0.00000	0.00000	0.00000	0.00000	0.00000	0.00000	0.00000	0.00000	0.00000	0.00000	0.00000	0.00000	0.00000	0.00018
	MNT	0.00423	0.00037	0.00211	0.00120	0.00413	0.00000	0.00000	0.00000	0.00000	0.00000	0.00000	0.00000	0.00000	0.00000	0.00000	0.00000	0.00000	0.00155
	SIN3A	0.00460	0.00595	0.00306	0.00216	0.00123	0.00000	0.00000	0.00000	0.00000	0.00000	0.00000	0.00000	0.00033	0.00005	0.00000	0.00000	0.00000	0.00209

Note: NSAF (normalized spectral abundance factor) represents the fraction of total immunoprecipitate that was represented by the number of peptides assigned to a given protein.
